# Recent Advances in Biomass-Based Materials for Oil Spill Cleanup

**DOI:** 10.3390/nano13030620

**Published:** 2023-02-03

**Authors:** Dan Ouyang, Xiaotian Lei, Honglei Zheng

**Affiliations:** 1 College of Textiles & Clothing, Qingdao University, Qingdao 266071, China; 2 Faculty of Information Science and Engineering, Ocean University of China, Qingdao 266100, China

**Keywords:** oil spill, biomass materials, oil absorption, oil water separation, hydrophobic

## Abstract

Oil spill on sea surfaces, which mainly produced by the oil leakage accident happened on tankers, offshore platforms, drilling rigs and wells, has bring irreversible damage to marine environments and ecosystems. Among various spill oil handling methods, using sorbents to absorb and recover spill oils is a perspective method because they are cost-effective and enable a high recovery and without secondary pollution to the ecosystem. Currently, sorbents based on biomass materials have aroused extensively attention thanks to their features of inexpensive, abundant, biodegradable, and sustainable. Herein, we comprehensively review the state-of-the-art development of biomass-based sorbents for spill oil cleanup in the recent five years. After briefly introducing the background, the basic theory and material characteristics for the separation of oil from water and the adsorption of oils is also presented. Various modification methods for biomass materials are summarized in section three. Section four discusses the recent progress of biomass as oil sorbents for oil spill cleanup, in which the emphasis is placed on the oil sorption capacity and the separation efficiency. Finally, the challenge and future development directions is outlined.

## 1. Introduction

Crude oil is a natural resource of crucial importance, playing an essential role for the development of human beings and society. The increasing development of economy and society makes it inevitable to extensively oil exploit and marine transport. However, frequently happened oil spill and oil leakage accidents, not only cause dramatically economic losses, but also brings major catastrophe to marine environments and ecosystem, and potentially further threatens human being life [1]. Consequently, oil spill cleanup has aroused widely attention both in the research community and the industry.

**Figure 1:**
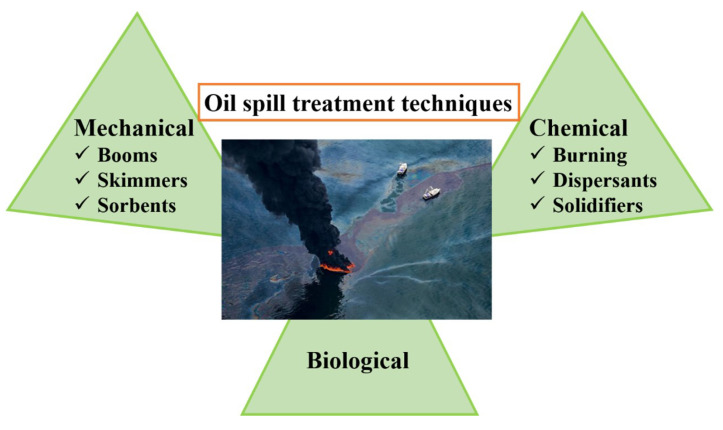
Three types of main techniques used for treating oil spills.

Currently, various methods and techniques have been developed to remove and recover oil spill on sea surfaces, which are mainly categorized into three types (Figure 1): (1) chemical method, (2) biological method, (3) mechanical method [2]. According to the real situation of oil spill-covered sea surfaces, these methods can be selected separately or in combination with each other. Chemical method involves in situ burning, dispersing with dispersants, solidification by solidifiers [3]. In situ burning can effectively remove oil spills in a relatively short time, but its produced toxic emissions during burning process, such as carbon dioxides, aromatic hydrocarbons, nitrogen oxides, etc. cause serious secondary pollution to the atmospheric environment. Dispersive and solidification methods is to remove oil contamination through interacting or reacting with oil by adopting surfactants or solidifiers, containing the utilization of toxic and unsustainable chemical materials. Biological method is to naturally biodegrade the oil into simple and non-toxic molecules based on the use of specific microorganisms (such as bacteria, algae, and fungi, etc.) [4]. It may bring the secondary pollution to the marine environment due to the introduction of exotic species. Mechanical method is to recover oil spills by using specialized equipment, such as the use of booms, skimmers, and sorbents, which is the least harmful approach to the environment comparing to chemical and biological methods [5,6]. Particularly, using oil sorbents to treat oil spills in water is the most attractive method among all methods because it is possible to completely collect and recover the oil in situ from water surfaces, environment-friendly, low-cost, and recyclable. Therefore, the increasing attention has been placed on the usage of sorbents for oil spill cleanup, especially for the development of efficient and eco-friendly oil sorbents.

Oil sorbents, typically, can be categorized as three major groups according to the material properties, namely inorganic materials, synthetic materials, and natural materials, for which each possesses their own merits and limitations (Table 1). Inorganic sorbents are usually natural or processed mineral or inorganic materials, for example, zeolites, silica, activated carbon, etc. [7,8,9,10,11]. These sorbents featuring with fine grain, porous, and highly dense, endows the advantages of the large surface area, chemical inertness, non-flammability, and easy availability, which makes it group of universal sorbents. However, they are difficult to recover as well as present poor separating efficiency and low oil sorption capacity, limiting their further application in oil/water separation. Synthetic sorbents are usually the polymer through artificial synthesis, including polypropylene, polyethylene, polystyrene, and polyurethane, etc. Even though they posse inherent hydrophobic properties, good reusability, and large sorption capacity, their potential environmental and ecological impact is not to be ignored due to the non-biodegradability. Natural sorbents, as like straw, cotton, and fruit peels, etc., featuring with the advantages of abundant, cheap, biodegradable, and eco-friendly. Despite the drawbacks of low capacity, poor hydrophobicity, and low selectivity, the researchers still endow numerous efforts to design efficient natural sorbents and promote their industrialization development.

Considering the huge demand of sorbents for tackling large area spill oils, so that the cost of sorbents should be affordable. It is highly desirable to seek green oil sorbents that are environmental-friendly and abundant. Biomass-based materials, derived from living organisms, such as plants, animals, and microorganisms, are one type of materials that are sustainable, biodegradable, and renewable. There are many kinds of biomass-based materials, which clarified and concluded from cellulose, chitosan, bio-carbon based materials, natural products in this review (Table 2). Cellulose is a long chain linear polysaccharide, extracted from plants, algae, tunicates and bacterial, etc. Chitosan is also a natural polysaccharide, extracted from crab shells, lobsters, etc. For bio-carbon based materials, they are prepared by the carbonization of raw biomass materials at high temperature treatment. Natural products defined as products that are acquired directly from the nature without extraction or treatment, which typically contain multiple components. Over the past few years, high-efficiency sorbents based on biomass materials have become the preferred choice for handling spill oils. There have issued numerous reviews on cellulose-based [12,13] and natural products-based [14,15,16] sorbents, covering their preparation, modification, and characteristics in detail. Furthermore, several reviews discussed the development of bio-based sorbents in terms of aerogels, membranes, gelators, and surfactants [17,18,19,20,21]. Notably, due to the poor oleophilic/hydrophobic properties of raw biomass materials, various modification methods have been developed to improve the hydrophobicity. Since the increasing development of sorbents in oil spill treatment, it’s well time to sum up the currently biomass-based oil sorbents from the modification method to the material properties. To our knowledge, the review on the modification and oil spill application of biomass-based sorbents covered cellulose, chitosan, bio-carbon, natural products, etc. is rare. The contents not only make researchers and readers quickly familiar with the development and the latest progress of biomass-based materials for spill oil cleanup, but also point out the existing problems and future researching direction in this field.

Herein, this review presents an overview of biomass-based sorbents for spill oils cleanup, in which both in their natural and modified forms within the recent five years. Firstly, we briefly introduce the basic theories and materials characteristics when the separation process of oil from water and the sorption process of oil (Section 2). Subsequently, different modification methods for improving the wettability (especially for the hydrophobicity) of biomass-based materials are presented in Section 3. Furthermore, various recently biomass-based sorbents for the spill oil treatment are classified and summarized in Section 4. Finally, a conclusion for this review paper and outlook for next-generation oil spill sorbents in the future, especially for biomass-based materials, are provided (Section 5).

## 2. Basic Theories and Material Characteristics

### 2.1. Oil/Water Separation

#### 2.1.1. Basic Theories

**Figure 2:**
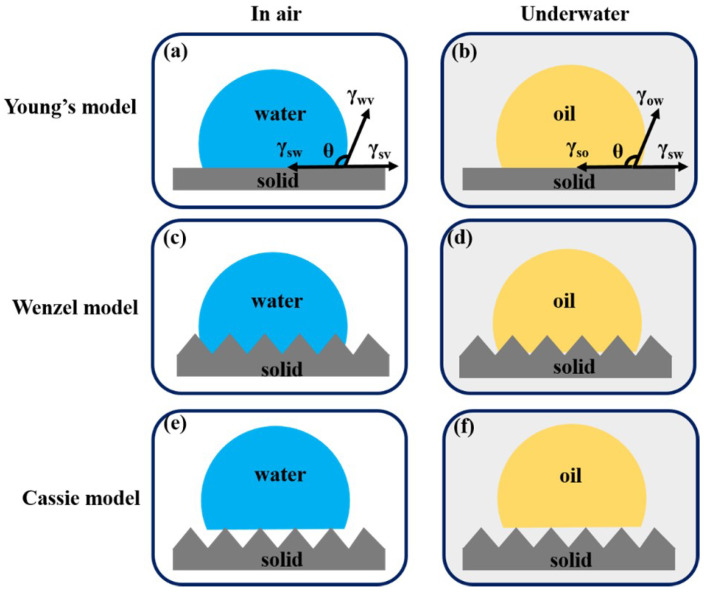
Schematic illustration of wetting mechanism. On the smooth surface (**a**) water droplet in air, and (**b**) oil droplets underwater. On the rough surface with the Wenzel state (**c**) water droplet in air, and (**d**) oil droplets underwater. On the rough surface with the Cassie-Baxter state (**e**) water droplet in air, and (**f**) oil droplets underwater.

The separation process of oil and water highly relates to the wettability of the liquid (water or oil) on the interface of the solid and air. When the wettability behavior occurs on an ideal smooth solid surface, the Young’s model is usually used to calculate the contact angle (θ${}_{y}$) [12] (Figure 2a):

(1) $$\cos\left(\theta_{y}\right)=\left(\gamma_{1}-\gamma_{2}\right)/\gamma_{3}$$

where γ${}_{1}$, γ${}_{2}$, and γ${}_{3}$ refer to the surface tension on the interface of the solid-vapor, solid-water, and water-vapor, respectively. Typically, the wettability can be assessed as: when 0° < θ${}_{y}$ < 90° is hydrophilic, 90° < θ${}_{y}$ < 180° is hydrophobic, θ${}_{y}$ < 10° is superhydrophilic, θ${}_{y}$ > 150° is superhydrophobic. Clearly, according to the Young’s equation, the surface wettability of materials can be tuned by changing the surface tension (surface energy) of the solid. However, by solely decreasing the surface energy cannot achieve a superhydrophobic surface [1,21]. To solve this problem, to design a rough structure on the solid substrate is proposed. This strategy combined with the adjusting of surface energy has been and broadly applied to achieve superhydrophobic surface.

When the wettability behavior occurs on a rough solid surface, the Wenzel model, the Cassie model, and the Wenzel and Cassie transient model are usually applied for determining the contact angle. When the liquid fully contacts with the rough surface and without any gap in the interface, the contact angle (θ${}_{w}$ ) calculates by using the following equation according to the Wenzel model [22] (Figure 2c):

(2) $$\cos\left(\theta_{w}\right)=R\cos\left(\theta_{y}\right)=R\left(\gamma_{1}-\gamma_{2}\right)/\gamma_{3}$$

where the roughness factor *(R)* is the ratio of the actual rough surface area to the geometrically projected area. Since the real area of rough surface is always larger than the projected area (*R* > 1), the contact angle on a rough surface is typically larger than that on a smooth surface. Thus, the wettability of materials can be adjusted by roughening the surface.

As shown in Figure 2e, if the liquid droplet partly contacts with the rough surface and there is air underneath a liquid droplet, the Cassie-Baxter model will be used to describe the contact angle (θ${}_{c}$ ) [23]:

(3) $$\cos\left(\theta_{c}\right)=R^{*}f\cos\left(\theta_{y}\right)+f-1$$

where the roughness factor ((R${}^{*}$)) represents the ratio of the actual wet part area to the projected area, which is typically lower than *R*. *f* represents the fraction of the wetted solid surface area account for the whole solid surface.

When external energy (such as the electrical field, vibration, and pressure, etc.) is applied, the contact angle (θ${}_{t}$) can be obtained by combing the Wenzel and Cassie equation according to the Wenzel and Cassie transient model [24]:

(4) $$\cos\left(\theta_{t}\right)=\left(f-1\right)\left(R-f\right)$$

Here, we just simply describe theories used in the designing of superhydrophobicity/superoleophilicity, superoleophobicity/superhydrophilicity, and underwater superhydrophobicity/superoleophilicity surface, respectively. More description about the relationship between surface wettability and surface roughness based on these models can be found in other recent review papers [12,13,15,16].

The different wettability for oil and water on the surface of materials make is possible for the separation of oil from water. In practical application of separation process, hydrophobic-oleophilic materials are always used. One phase from the mixture of oil and water can pass through the separating materials due to the pressure different between two side of this material. Typically, pore size and intrusion pressure are two major factors relating to the separtion process. The intrusion pressure (▵P ) is defined as the maximum static pressure withstand by the separating material, which highly relates to the advancing angle and pore size according to the following equation [25],

(5) $$▵P=2\gamma /R=l\gamma \cos(\theta_{a})/A$$

where γ refers to the surface tension, *R* is the radius of the meniscus, *l* represents the perimeter of pores, θ${}_{a}$ refers to the advancing contact angle, and *A* is the pore size.

The continuous phase flow could be expressed by Hagen-Poiseuille equation [26]:

(6) $$J=r_{p}^{2}▵P\epsilon /8d\mu \tau$$

where *J* refers to the permeation flux, ▵P is the intrusion pressure, ϵ is the porosity, *d* is the material thickness, μ represents the liquid viscosity, and τ refers to the tortuosity. According to the above equation, an ideal separtion material should consider the following three aspects: (1) opposite affinity to water and oil; (2) suitable pore size for the selective permeation; (3) as much porosity as possible [27].

#### 2.1.2. The Material Characteristics for the Separation of Oil and Water

Typically, ideal materials for separating oil from water own some criteria (Table 3), involving high separating efficiency, mechanically elasticity, chemical stability for corrosive environments (such as acid, base, salts, etc.), inexpensive, and ecosystem friendliness, and so forth [14].

According to the wetting theories discussed above, it is easy to understand that the surface chemical composition and surface roughness are two important factors to influence the separation efficiency. First, the wettability and solid/liquid adhesion behavior can be governed by the chemical composition for achieving various surface energies. Furthermore, the morphological and scale of structures roughness on the surface are also vital for achieving suitable hydrophobicity so that efficient separating the oil and water. Therefore, design of a material with a suitable curvature or wettability gradient surface is a significant strategy to enhance the separation performance. The efficient strategy for designing oil-water separating materials is to construct a rough surface with suitable hydrophobic.

Besides, the selective is another characteristic for high-performance separating materials. For effective isolate oil from water, the requirement for the materials is that there is a high positive breakthrough pressure to the oil phase and a high blocking pressure to the water phase.

### 2.2. Oil Sorption

#### 2.2.1. Basic Theories

**Figure 3:**
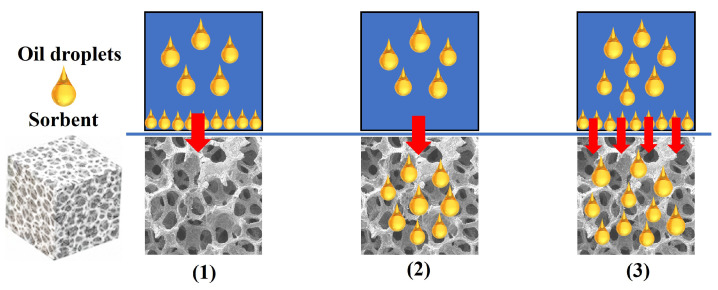
Schematic diagram of oil absorption process. (1) oil droplets accumulate on the surface of sorbents via weak interaction forces; (2) oil droplets penetrate the intermolecular of sorbents under the capillary forces; (3) both the surface accumulation and intermolecular penetration exist.

The oil sorption process can be operated by the form of intermolecular penetration, accumulation on the surface, or a combination of both (Figure 3). On the one hand, the oil prefers to penetrate the gap spaces or pores of sorption materials under the capillary forces. On the other hand, the oil will retain or accumulated on the surface of sorbents via hydrogen bonding, steric interactions, or other weak interaction forces [20]. In a real situation, the accumulation and intermolecular penetration are coexistent in the whole oil sorption process. In other words, the oil will accumulate on the material surface via weak interaction forces and simultaneously penetrate to the internal holes of sorbent materials via the capillary effect.

Even though the understanding of sorption mechanisms is necessary, the uptake kinetics is more essential for achieving efficient oil absorption once the oil sorption process is working. When the oil is favorably penetrated the sorbent materials, the transport of oil into the voids of sorbents is governed by the capillary forces, which can be promoted by pressure and gravitational forces. When the oil prefers to retain on the surface of sorbents in the sorption process, it is typically controlled by the attraction between the outer surface of sorbents and the oil, which can be driven by physical and chemical interactions (such as hydrogen bonding, van der Waals forces, etc.). That’s means that the surface chemical state and morphology play a crucial role for the oil adsorption process. Moreover, the uptake kinetics, even oil sorption mechanisms is highly dependent on the oil properties (including viscosity, polarity, and molecular size, etc.). In a successful sorption process, firstly, sorbents should have the required critical surface tension value, which can be modified with physical and chemical surface treatment. And then the uptake kinetics is considered for achieving high oil sorption capacity from the aspect of the porosity of sorbents, oil properties, and so on.

#### 2.2.2. The Material Characteristics for Oil Adsorption

As suitable oil spill sorbents, it prefers to possess certain properties (Table 3), including hydrophobicity and oleophilicity, high sorption capacity, buoyancy, recyclability, environmental-friendly, stability, and so on.

To adsorb the oil from the water efficiently, sorbents should be oleophilicity and hydrophobicity. Thus, the wettability is one essential property for oil sorption materials, which highly depending on the morphology and the surface chemical state of material. In terms of morphology, the porosity of materials is vital parameter for achieving high oil adsorption capacity since the oil prefers to penetrate the pores through capillary forces. For the surface chemical composition, it dominates the wettability and solid/liquid adhesion interaction. When the oil is retained and accumulated on the surfaces by weak interaction forces (such as van der Waals forces, polarity, and other physical and chemical interactions, etc.), the surface wettability of sorbents can be tuned by adjusting or modifying the surface chemical.

To avoiding secondary pollution during oil separation and recovery process, developing recyclable and biodegradable absorbents are also essential consideration. The recyclability or reusability of biomass-based materials after the process of absorbing oil and oil recovered are of great significance for energy conservation and environmental protection. There are many methods have been investigated to recycle biomass materials, namely mechanical squeezing, heating, solvent extraction, burning, and vacuum filtration [43,44]. By controlling the heating temperature to the boiling point of the absorbates, the solvent-saturated sorbents could be recycled [45,46,47]. However, it is limited to the low boiling point solvents (such as hexane, chloroform, etc.) in case to preventing the utilization of high temperature. Solvent extraction is to immerse the saturated sorbents into suitable solvent for the extraction of oils and the reusability of sorbents [48,49]. However, its shortcoming of complicated, incomplete, and costly is concerned. Flammable gasoline involved in spill oil can be removed by burning, which is the commonly used approach for recycling carbon aerogel [43,50]. However, the secondary pollution produced during the burning process is the major issue for limiting its practical application. For vacuum filtration, the high pressure used in the whole process make it trouble for high-viscosity solvents, and complex equipment and high energy consumption are also concerned. Comparing to heating, extraction, burning, and vacuum filtration, squeezing approach is more popular due to its unique features of green, simple, less energy consumption, economic, and available for practical application [29,51,52,53]. Moreover, it is typically used for high viscosity oil. In summary, for biodegradable biomass-based materials, the recyclability is a crucial feature considerated in practical application and environmental protection, which is usually considerate for the material design. Notably, the recycling performance obtained by using different methods may diverse.

## 3. Modification Method for Biomass-Based Materials

**Figure 4:**
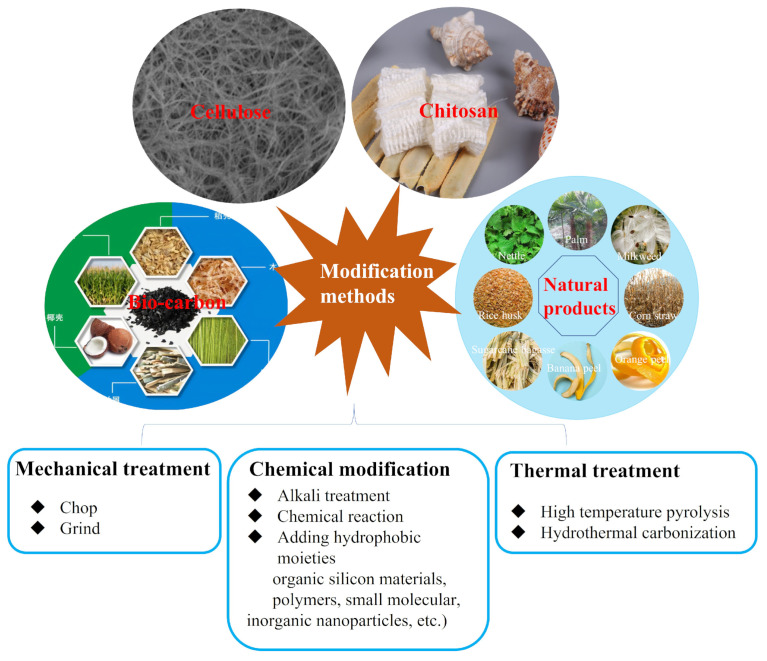
Modification methods for biomass materials.

Most raw biomass-based materials, such as celluloses, chitosan, natural products, etc. are typically amphiphilic, which tend to absorb the water and oil simultaneously. In order to change the surface wettability (improve the hydrophobicity), various surface modification on biomass materials have been developed, which may be clarified as three types (mechanical treatment, thermal treatment, chemical modification) herein for the discussion, as showing in Figure 4.

### 3.1. Mechanical Treatment

Mechanical treatment, for example chop, grind, etc. is a simple and traditional method to treat some natural products (such as corn, straw, etc.). Previous studies have issued that that grinded materials show higher oil sorption capacity than those untreated materials [17]. That is because the smaller particles after grinded can provide more contacting surface and binding sites and make the oil more accessible and accumulation. Typically, as the sorbent size decreases, the surface area per unit mass increases and more binding sites are available for oil to be adsorbed, therefore, adsorption capacity will be increased. For example, Bayat et al. investigated the effect of particle size of bagasse on the oil adsorption capacity and found that the adsorption capacity of 18 to 45 mesh bagasse (5–6 g g${}^{-1}$) was larger than that of 14 to 18 mesh (3–5 g g${}^{-1}$) [54]. The same observation was also obtained by Ibrahim et al.’s study. The oil adsorption capacity of the surfactant modified barley straw (SMBS) with different particle size of <0.50, 0.50–1.18, and 1.18–1.4 mm was 92.0 ± 0.5, 83.2 ± 1.9, and 80.6 ± 1.6 mg g${}^{-1}$, respectively, indicating the higher adsorption capacity for the smaller sorbent size [55]. However, the effect of simple mechanical treatment is limited for improving the oil sorption capacity. On the one hand, the scale of sample size reduction is micron or above, the increase of surface area is limited. On the other hand, there is no contribution to the hydrophobicity (or lipophilicity) improvement of sorbents. While the hydrophobicity is the driving force of oil sorption and play important role in the oil adsorption capacity [56].

### 3.2. Thermal Treatment

Typically, low-temperature treatment can just remove the surface contaminants, while high-temperature pyrolysis can lead to the carbonization of materials. Sorbents treated by high-temperature pyrolysis presented significantly enhanced oil sorption capacity and selectivity of oil and water [18]. Zhu et al. study the change of internal morphology and chemical composition of carbonized pomelo peels under different pyrolyzing temperature (600–800 °C). The found that the weight gain (defined as the weight of absorbed substance per unit weight of the aerogel) of the prepared porous carbon aerogel decreased from 3425% to 2647% when the calcination temperature was increased from 600 °C to 800 °C. That’s because there is more defect structure and smaller pores, as well as less hydrophilic groups on the materials surface as the increasing of the carbonization temperature [19]. Similarly, Gheriany’s study found that the structure of orange peels became rough with increased porosity after 500 °C thermal decomposition comparing with the originally smooth and homogeneous structure [57]. The oil sorption capacity of thermal modified orange peels at 500 °C was enhanced 45% than that of original orange peels (5.23 g g${}^{-1}$ after 60 min). They supposed that the material decomposition and sorption of vapors during pyrolysis may produce more pores. It is easily concluded that the rough and porous structure constructed by high-temperature pyrolysis contribute to the improved oil sorption capacity.

Hydrothermal treatment as another thermal treatment can improve the hydrophobicity by enhancing the carbon content of natural materials [58]. Comparing to conventional high-temperature pyrolysis, hydrothermal treatment is more available and simpler because they avoid the complex and time-consuming heating process as well as energy loss [59]. Hydrothermal carbonization (HTC) is a promising method for the treatment of biomass materials because it requires less energy and produces various micro- or nanostructure under economical and simple approach [60]. The detail process is to put raw materials into a Teflon-lined stainless-steel autoclave and sealed for hydrothermal carbonization. The sealed autoclave was placed in an oven and performed at mild or high temperature. The HTC under moderate temperature is preferable due to more easily adjustable morphology for hydrochar and low energy consumption [61]. However, when applying high temperature, some interesting carbon structures like tubular, nanotubes, and microspheres, etc. could be obtained, which is exhibits high adsorption capacity [62]. Wang et al. studied the oil adsorption performance of zinc oxide nanoneedle decorated kapok fiber obtained by one-step hydrothermal method. The fabricated fibers show high oil sorption capacities up to 29.5–52.8 g g${}^{-1}$[58]. According to the investigation of Zhu’s group, the fabricated porous carbon aerogel by the simple HTC displayed the sorption capacity ranged from 5 to 36 g g${}^{-1}$ for different organic solvents and oils [19]. In Li’s study, the winter melon carbon aerogel prepared by HTC showed an adsorption capacity of 25 g g${}^{-1}$ [63].

### 3.3. Chemical Modification Treatment

Due to the poor hydrophobicity and buoyancy of most biomass-based materials, proper decoration and modification are of great significance. To enhance the hydrophobicity and oil sorption capacity of biomass-based materials, a variety of chemical modification approaches have been reported in literature, such as alkali treatment with hot or cold alkali solution [64,65], converting hydrophilic groups to oleophilic groups by chemical reaction [10,66,67,68], the addition of hydrophobic moieties [69]. Among them, to add hydrophobic moieties by surface chemical modification is the most used technique, and it highly relies on the chemical structure of biomass-based materials. Surface modification of biomass-based materials by using different chemicals will provide suitable surface wettability so that they can absorb oil and repel water. In this paper, all modification materials are roughly grouped as three types of organic materials, inorganics materials, and others.

#### 3.3.1. Organic Materials

Silicon based materials: Thanks to the flexible backbone and weak molecular interaction, organic silicon materials containing hydrophobic functional groups (such as long organic chains) and active groups (such as silicon-oxygen) are broadly applied to lower the surface energy of sorbents. Active groups of organic silicon conduct condensation reaction with hydroxyl groups of biomass materials, and then its long-chain alky group (the hydrophobic group) will be successfully introduced to the surface of materials and achieve hydrophobicity of materials. Due to their high elasticity and hydrophobicity, various types of organic silicon materials have been extensively researched to modify biomass-based materials, for example methyltrimethoxysilane (MTMS), trimethylchlorosilane (TMCS), perfluorodecyltriethoxysilane (PDTS), polydimethylsiloxane (PDMS), etc.

**Figure 5:**
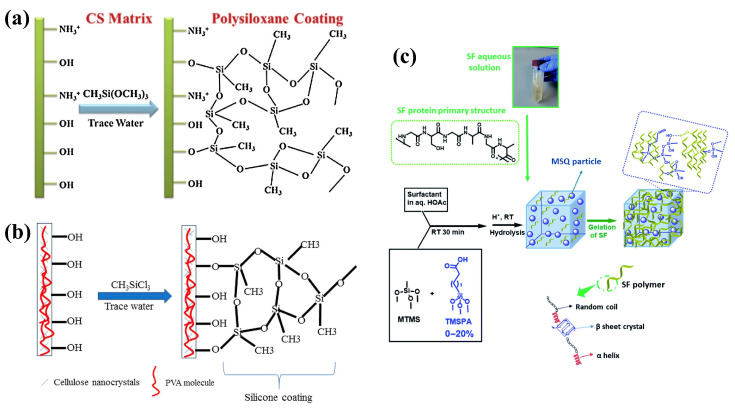
(**a**) Schematic illustration of the possible chemical reaction between MTMS and hydroxyl groups on the surface of chitosan. Reproduced with permission [76]. Copyright 2020, Elsevier Ltd. (**b**) Reaction scheme of MTCS with hydroxyl groups on the surface of the aerogel. Reproduced with permission [87]. Copyright 2019, American Chemical Society. (**c**) Synthesis of PMSQ-SF hybrid aerogels by using silicon-based coupling agent. Reproduced with permission [97]. Copyright 2018, Royal Society of Chemistry.

Silicon-oxygen (Si-O) functional group is one type of active groups, and many organosilicons with multiple Si-O groups have been adopted for hydrophobic treatment of biomass-based materials. MTMS is the most frequent used organosilicon for hydrophobic modification as it is very simple and cheap. Cellulose nanofiber [70] and pineapple fiber [71] treated with MTMS for improving the hydrophobicity have been studied. Aerogels based on diverse biomass materials were also modified with MTMS to obtain hydrophobicity [72,73,74]. Oliveira et al. made hydrophobic Pinus elliotill-derived aerogel by vapor-phase deposition of MTMS [75]. By the self-polymerization reaction of MTMS, Yi et al. formed a cross-linked superhydrophobic polymer on the chitosan aerogel [76]. The possible reaction between MTMS and the chitosan skeleton are illustrated in Figure 5a. Yi et al. used MTMS as a silanization agent to improve the hydrophobicity of lignin-polyvinyl alcohol aerogel via chemical vapor deposition reaction [77]. To further improve the hydrophobicity, the researchers tried to change the short methyl chain to longer alkyl chain. Dodecyltriethoxysilane [78] Hexadecyltrimethoxysilane (HDTMS) [52], octadecyltrimethoxysilane (OTMS) [49] have been utilized for the hydrophobic treatment. Depositing HDTMS on cellulose nanofibers and cellulose sponges via chemical vapor deposition (CVD) method can successfully achieve hydrophobic surface [79,80]. Laitine et al. tried two types of silylation agents (MTMS and HDTMS) to improve the hydrophobicity [81]. Zhang et al. replaced the -OH functional groups in titanium oxide (TiO${}_{2}$) NP by long chain alkyl group in OTMS and obtained hydrophobic TiO${}_{2}$ NPs. Furthermore, organosiloxanes containing other functional groups (such as vinyl, amino group, etc.) may bring other reaction sites, have also been investigated for surface modification. By treated with vinyl-trimethoxysiliane, Wu et al. achieved silylated cellulosed sponge with underwater oleophobicity [82]. Yun et al. obtained hydrophobic composite aerogels by coated with hexamethyldisilazane via chemical vapor deposition [83]. The hydrophobic chitosan-coated magnetic nanoparticles were achieved by depositing with silica and 3-aminopropyltriethoxysilane [84].

Organochlorosilanes is another hydrophobic treating agent. TMCS possesses the advantages of low cost and simple deposition process, which has been widely chosen as the coating agent to fabricating hydrophobic and oleophilic surface. Xu et al. adopted TMCS to modify cellulose nanofiber-based aerogel for adjusting its hydrophilicity [85]. Fan et al. also treated cellulose-based aerogels with TMCS for achieving hydrophobic properties [86]. To increase the number of chlorine element in organochlorosilanes, the hydrophobicity may be significantly enhanced due to the increased reaction sites and then increased hydrophobic groups. Methyltrichlorosilane (MTCS) was applied for hydrophobic modification of cellulose nanocrystals-based aerogels, as shown in Figure 5b [87]. Xu et al. also coated MTCS on the surface of aerogels via the CVD method for improving the hydrophobicity [37]. Other aerogels, such as gelation aerogels [88] and chitosan aerogel [89], were also treated with MTCS to further improved the hydrophobicity of materials. Similar molecular, trichlori(octyl)silane, was adopted by Deuber et al. to modify the amphiphilic framework of the aerogel [90].

As we all known, fluorine containing materials exhibits hydrophobicity, and the more fluorine on the side chain, the stronger hydrophobicity. A variety of fluorine containg organosilican have been applied for the reduction of surface energy and the achievement of superhydrophobicity, such as PDTS [91], perfluorooctyltriethoxysilane (PFOS) [92], and (Heptadecafluoro-1,1,2,2-tetradecyl) trimethoxysilane (HDFTD-TMOS), etc. In Lu’s work, the long-chain silane, PFOS was also been utilized for treating the prepared aerogel to obtain superhydrophobic [93]. Xu et al. deposited HDFTD-TMOS on the surface of corn straw fibers and found the reduced surface energy and improved hydrophobicity [94]. 1H, 1H, 2H, 2H-perfluorodecyltriclorosilane as a hydrophobic fluoroalkylsilane, has been used by Zhang et al. for surface hydrophobic modification [95].

Crosslinked polysilanes were also applied for surface modification to obtain hydrophobic materials. By forming polysiloxane on the surface of the spongy aerogel, Li et al. achieved a satisfactory hydrophobicity [96]. As shown in Figure 5c, Maleki et al. applied polymethylsilsesquioxane aerogels derived from the trifunctional MTMS to improve the hydrophobicity and mechanical properties [97]. Take advantage of the low surface energy of PDMS, Zhang et al. prepared a porous super-hydrophobic sponge [98]. Later, Wang et al. obtained the super-hydrophobic property by using a PDMS/curing agent system [99].

Polymers: Polymer with hydrophobic-oleophilic properties could be used as low-surface-energy materials for surface modification of biomass-based materials. Polythiophene has been selected by Durgadevi et al. to make a surface hydrophobic [100]. It’s because a series of aromatic thiophene ring on the polymer backbone makes it insoluble or hard to dissolve in common solvents (such as water, ethanol, even benzene, etc.). Ma et al. treated fibrous membranes with fluorinated polybenzoxazine [101]. In another work, Shang et al. obtained the a novel biomass-based benzoxazine (C-aPOSS) derived from cardanol and amino-propyllsobutyl [102]. The C-aPOSS was polymerized on the cotton fiber surface through thermal treatment ring-opening reaction to obtain the superhydrophobic. Wang et al. tried polyhedral oligometric silsesquioxane based compound as hydrophobic treating agent [103]. Polyhemiaminal was used to prepare wettability-switchable bacterial cellulose aerogels [67]. Poly (N, N-dimethylaino-2ethyl methacrylate) polymer was also used as hydrophobic treating agent to modify aerogels [104,105].

Small molecular: A few organic small molecular with long aliphatic chains and polar groups (such as acyl chloride, organic acid, thiol, etc.) have also been used for achieving low-surface-energy materials. On one hand, the polar group of small molecular can be attached to biomass materials via chemical reaction or weak molecular interaction. On the other hand, the long and water-insoluble nonpolar chain can make sure the hydrophobicity of materials.

**Figure 6:**
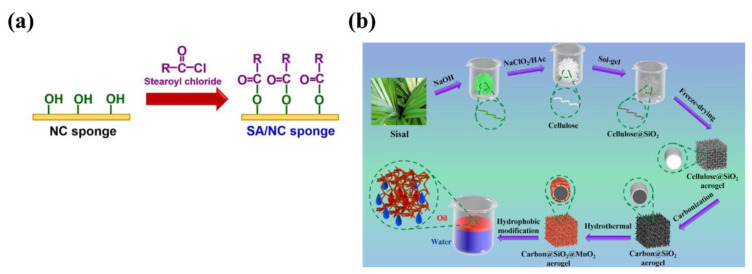
(**a**) Schematic of surface modification on NC sponge by stearoyl chloride (R-C${}_{17}$H${}_{35}$). Reproduced with permission [36]. Copyright 2018, Elsevier Ltd. (**b**) Schematic illustration of fabrication of the modified HBCSM aerogel. Reproduced with permission [115]. Copyright 2019, American Chemical Society.

As reported by researchers, aliphatic acyl chlorides are promising hydrophobic modification agent that can be used to achieve hydrophobization by covalent surface grafting [48]. By treating with stearoyl chloride for nanocellulose sponge (as shown in Figure 6a), Phanthong et al. successfully improved the hydrophobicity of original sponge [36]. Chhajed et al. prepared a super-hydrophobic composite aerogel by dipping into the stearoyl chloride solution [106]. Stearic acid containing 18-carbon chain is also investigated as modification agent to reduce cellulose surface energy [107]. Another organic acid with strong lipophilicity, oleic acid (OA), has also been used as a hydrophobic treating material for building unique interfaces. Gu et al. added OA into cellulose to enhance the hydrophobicity of cellulose [108]. Thanks to the hydrophobicity of long chain and the polarity of Sulfur-Hydrogen group, several long chain thiols have been adopted for surface modification. Dodecanethiol [59] octadecanethiol [109], and other thiols as hydrophobic modification agents for different biomass materials have been investigated. Liu et al. modified porous sponge with octadecanethiol and silicon dioxide (SiO${}_{2}$) nanoparticles [41]. Cao et al. applied 1H,1H, 2H, 2H-perflurodecanethiol to create superhydrophobic surface [110]. Besides, Gao et al. endowed the cellulose nanocrystals with hydrophobicity by modified with octadecylamine molecules [111]. By treating hydrophilic hydroxyl containing materials with blocked diisocyanate to obtain hydrophobic properties has been successfully conducted by Souza et al. [112]. Xu et al. conducted hydrophobic modification with epoxidized soybean oil [113].

#### 3.3.2. Inorganic Materials

Nano inorganic materials featuring with small size, various geometric structure, large specific surface area and high surface energy can be used to construct rough surface on biomass materials. However, due to their inherent polarity and hydrophilicity, it usually needs to further improve their hydrophobicity from synthesis approach or combining other materials. Zhang et al. synthesized a superhydrophobic TiO${}_{2}$ nanoparticles and coated on cellulose sponges to achieve suitable hydrophobic properties [49]. Yang et al. constructed micro-nanoscale hierarchical structures for enhancing the wettability by coating with SiO${}_{2}$ and TiO${}_{2}$ microspheres [114]. The strategy of in-situ growth of SiO${}_{2}$@ manganese dioxide (MnO${}_{2}$) nanostructure on the surface of carboned aerogel (named as HBCSM shown in Figure 6b) has been successfully adopted by Yuan et al. to improve the surface roughness and hydrophobic properties [115]. Simply depositing copper nanoparticles on the surface of cellulose fibers can also enhance the hydrophobicity of material [116]. Graphene and its derivatives are two-dimensional nanomaterials, which can enhance the hydrophobic properties when mixing with biomass materials. Zhang et al. reported that graphene added into a polymer matrix can enhance the hydrophobicity and oleophilicity [95]. In Meng’s study, graphene oxide was introduced into the lignin-based carbon aerogel, which contributes to further improve the hydrophobicity and makes it superhydrophobicity [117].

#### 3.3.3. Others

Salts can also be utilized on certain biomass materials to modify their surface wettability. Sun et al. reported to use sodium periodate and sodium sulfite to modify a cellulose nanofiber aerogel [118]. Jiang et al. attached zwitterionic compounds on a composite aerogel for achieving superhydrophilicity, superoleophilicty, and underwater superolelophobicity as well as underoil superhydrophobicity [119]. Dan et al. dramatically improved the hydrophobicity of Enteromorpha aerogels by introducing ammonium dihydrogen phosphate (NH${}_{4}$H${}_{2}$PO${}_{4}$) as a modifier thanks to the removed oxygenated groups and enhanced roughness on the surface of the Enteromorpha [120].

For most organic materials, such as silicon-based materials, Fluorine-containing compounds, and so on, their toxicity and cost are concerns. silicone materials. Even though inexpensive inorganic nanoparticle materials, like ZnO, SiO${}_{2}$, etc. can modify the surface properties of biomass-based materials, their impacts on the ecosystem, even for human beings, are still uncertain. Moreover, the synthesis process and conditions of these nanostructured inorganic materials are typically sophisticate. Importantly, most of the reported chemical modification materials are not sustainable and without using renewable resources.

### 3.4. Environmentally Friendly Modifications

Even though the above-mentioned technologies (mechanical, thermal, and chemical) have varying effectiveness and durability in tunning the hydrophobicity and oleophilicity of biomass materials, the sustainability and ecological concerns and cost limit their practical application. Therefore, to explore environmental-friendly and ecologically sustainable methods to achieve hydrophobic modification of biomass materials have drawn increasing attention. Lignin as one biomass resources, contains hydrophobic phenyl propane skeleton in its three-dimensional aromatic polymer main chain. Thus, efforts have been conducted to exploit lignin as hydrophobic feedstock in oil sorbents. Yang et al. proposed a lignin-based hydrophobic coating for designing a “green” oil sorbent [121]. Chen et al. incorporated hydrophobic lignin additive into porous network of graphene aerogels for the fabrication of efficient and recyclable absorbents [46]. Ferreira et al. used lignin as a hydrophobizing agent to pretreat the cellulose fibers for achieving lightweight and hydrophobic foam materials [122]. Natural waxes are another widely used hydrophobic surface modification materials. By adopting beeswax to provide low surface energy, Zhang et al. demonstrated a biomass-based porous materials with super-hydrophobicity [123]. Lin’s group utilized carnauba wax and beeswax as hydrophobic modifier to develop an environmentally friendly all-biomass aerogel [124]. Others, like natural rubber, polymeric epoxidized soybean oil has also been investigated as hydrophobic component for achieving eco-friendly and hydrophobic materials [29,113].

Except for materials, there is limited studies relating to surface processing technologies for surface modification of biomass materials. For example, Mahamoud studied the influence of microwave technique on the sorption capacity of flax fibers. The contribution to the increased oil sorption capacity is induced by the change of surface topography not the surface wettability [68].

Although many efforts have been done for finding ecologically sustainable alternatives of the commonly used chemical modifications, environmental-friendly methods for surface modification of biomass materials are still urgently needed.

## 4. Types of Biomass-Based Adsorption Materials

As discussed in Section 2, for a successful oil/water separation, the wettability of biomass-based materials is of great significance. That’s means that the critical surface tension of the sorbent should be larger than the surface tension of the oil and smaller than that of the water. Typically, to gain the desired critical surface tension of sorbents, suitable chemical and physical surface modification is needed. Moreover, the mechanism is strongly depended on the oil properties, such as viscosity, molecular size, and polarity, and so forth. For various biomass-based materials, the mechanism maybe the same or different, which depends on the material chemical and physical properties. The mechanism of oil/water separation by using cellulose and chitosan and their composite are strongly depends on the surface chemical state, morphology, and another component. The oil maybe favorable retain on the surface, or penetrate the intermolecular of materials, or both of them. Since natural products are mostly cellulose-containing materials, there is the same situation as that of cellulose in terms of the mechanism. Bio-carbon based materials and various aerogels (such as cellulose, chitosan, bio-carbon based aerogels, and others) have high porose and large specific surface area. Others, like lignin and alginate, can also form 3D network structure. For these biomass-based materials, the penetration of oil into the sorbent material dominates, which is driven by the capillary forces.

### 4.1. Cellulose

Cellulose is one of the most abundant polysaccharides and can degrade easily. Numerous reviews on cellulose and its derivatives have been released, relating to synthesis approaches, material characterization, hydrophobic modification, performance, and industrialized application, which offers clearer recognize about this material [20,125,126]. Thanks to the advantages of easily available, abundant, renewable, biodegradable, easily modification, cellulose-based materials have been extensively applied for spill oil treatment (Table 4).

Cellulose nanocrystals (CNCs): Cellulose nanocrystals typically possess rod-like nanostructure, and their morphologies and dimensions highly depend on the cellulose source and preparation methods. Moreover, the CNCs with nano-scale size prefer to aggregate together and then generate irregular particle with rough surface morphology.

There are numerous hydroxyl groups hanging out the surface of CNCs, leading to their highly hydrophilic and oleophobic. By take advantage of the oil-repelling properties of CNCs, Wu et al. fabricated CNCs decorated nanopapers and nanocoatings with ultralow crude oil adhesive forces [28]. Furthermore, the obtained nanopaper and nanocoating both exhibited an underwater oil contact angel larger than 150°, which were successfully adopted for efficiently separating oils from oil-in-water emulsion and oil/water mixtures.

**Figure 7:**
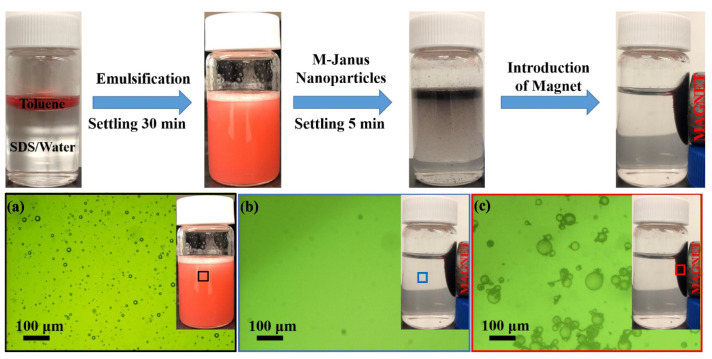
Phase separation process of SDS stabilized toluene-in-water emulsions with the addition of M-Janus nanoparticles under the external magnetic field (top row), and micrographs of the SDS stabilized toluene-in-water emulsion droplets (down row). (**a**) Stable tiny toluene droplets before the phase separation. (**b**) Clear water phase after the phase separation and (**c**) M-Janus nanoparticle-tagged toluene droplets after the phase separation. The scale bars are 100 µm. Reproduced with permission [128]. Copyright 2019, Elsevier Ltd.

Owing to the properties of rough morphology and easily modification, modified CNCs are commonly applied as coating materials for adjusting the wettability, and then applied for recovering crude oils from water. Superhydrophobic CNC paints were prepared by Huang et al. via a simple solvent exchange method, which could spray on the steel mesh for achieving the superhydrophobicity [127]. By absorption of hydrophobic ethyl cellulose and hydrophilic carboxymethyl cellulose on the opposite sides of magnetite (Fe${}_{3}$O${}_{4}$) nanoparticles, He et al. successfully prepared magnetically responsive and interracially active Janus nanoparticles (as shown in Figure 7) [128]. These Janus nanoparticles exhibited excellent capability and high efficiency in separating emulsified water form water-in-crude oil emulsions and the oil from oily wastewaters under an external magnetic field. Prathap et al. developed a novel and eco-friendly organogelator-cellulose composite materials by combining hydrophilic cellulose nanocrystal and amphiphilic gelator, which displayed high selectivity, efficiency, and instantaneous for the retrieving of oil from emulsions [129].

Cellulose nanofibers (CNF): Cellulose nanofibers are a long and twisted cellulose matrix, containing isolated and clustered cellulose fibers [130]. CNFs and their derives have been extensively investigated in oil sorption and oil/water separation thanks to the distinctive features of good durability and high specific surface area [131]. A hydrophobic nanocellulose foam prepared by lyophilization pure nanocellulose and hydrophobic modification exhibited high oil absorption capacity (22–55 times higher than its dry weight) and separation efficiency [36].

**Figure 8:**
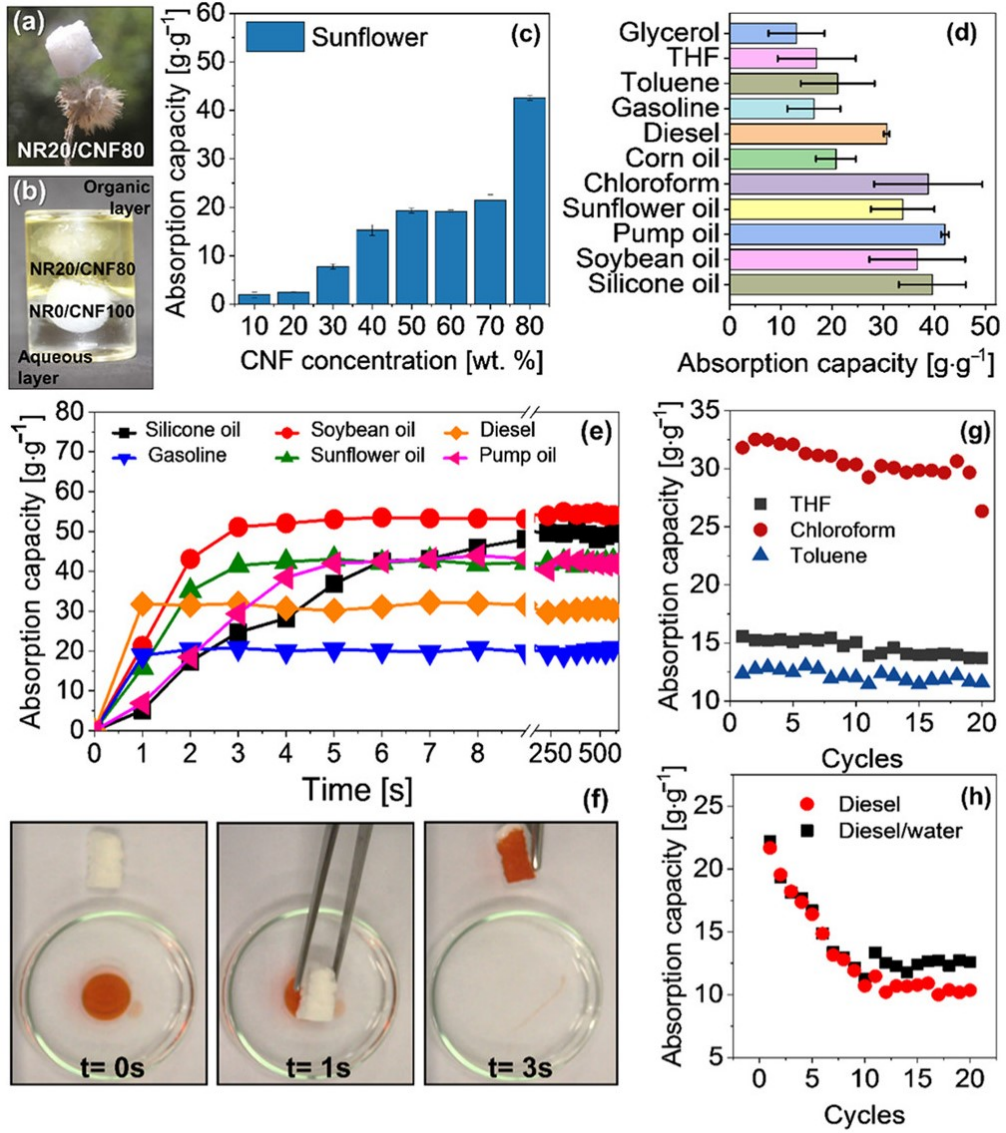
Absorption capacity and reusability of NR/CNF foams. Conceptual test: digital photographs showing (**a**) ultralightweight foam placed on Taraxacum sp. and (**b**) hydrophobic affinity (NR20/CNF80, top) and hydrophilicity (NR0/CNF100, bottom). (**c**) Sunflower oil absorption assay for different CNF contents (10–80 wt%). (**d**) Absorption capacity of NR20/CNF80 foams for different types of oils and organic solvents. (**e**) Kinetics for different oils and organic solvents for NR20/CNF80 foams from 0 to 600 s (the x-axis has a break between 10 and 100 s since the plateaus have reached). (**f**) Representative pictures of absorption kinetics for soy oil (orange). (**g**) Representative curves of repeated cycles (0–20) for mass-based absorption capacity of the NR20/CNF80 foams at room temperature for organic solvents: THF, chloroform, and toluene. (**h**) Representative curves of the reusability test for diesel oil and diesel/water mixture. Reproduced with permission [29]. Copyright 2020, American Chemical Society.

Since they can solve the hydrophilic property of CNFs and make full use of the advantages of other materials, combining CNFs with other materials as composite oil sorption material have been widely studied. By combing polyimide, fluorinated polybenzoxazine, and silicon nanoparticles with cellulose acetate, Ma et al. fabricated a separating membrane with high flux and separation efficiency [101]. Calcagnile et al. prepared a novel composite sorbent material by using cellulose three-dimensional fibrous as scaffold and decorating with expanded graphite flakes, which displayed high selectivity for various oils and organic solvents [107]. As shown in Figure 8, Lorevice et al. designed an eco-friendly and hydrophobic foams by evenly distributing natural rubber (NR) throughout the surface of CNFs [29]. The obtained composite foam presented excellent sorption capacity and speed (above 50 g g ${}^{-1}$ in 3 s), and great reusability for various organic solvents and oils. Take fully advantage of the synergistic effect of cellulose and chitosan, Hardian et al. fabricated an oil and solvent-resistant nanofiltration membrane with superior separation efficiency and chemical stability [132]. Halima et al. adopted two kinds of modified CNFs to create a hydrophilicity and underwater oleophobicity cellulose sponge, which shows high separation efficiency (99%) by gravitational force alone [133].

Cellulose-based aerogels: Aerogel featured with porous structure, ultra-lightweight, small density, and large specific surface area, which have been widely applied as sorbents to recover the spill oil form water. Considering their respective unique properties of aerogels and cellulose, designing cellulose aerogel as a new category oil sorbents have intensively aroused researchers’ attention [134].

Different cellulose-containing substances have been utilized for the preparation of cellulose-based aerogel, for example Pinus elliotii [75], canola straw [52], bamboo leaves [135], Eichhornia crassipes [136], raw cotton [137], recycled box board and recycled milk-container board [81], etc. The specific mass, density, porosity, adsorption capacity for these cellulose aerogels is different, which is highly depends on the cellulose resource, the preparation approach, and the modification method. The cellulose aerogel based on Eichhornia crassipes synthesized by Yin et al. exhibited an oil/solvent sorption capacity ranging from 60.33 to 152.21 g g ${}^{-1}$, and excellent reusability [136]. Wang et al. prepared superhydrobobic cellulose aerogel using raw cotton fiber, which presented superhydrophobic property (>154°) and great sorption capability for various organic solvents and oils (19.8–41.5 times its own weight.) [137]. a dual superlyophobic lignocellulosic fiber-based aerogel was successfully prepared by Kang et al., which can easily change from amphotericity in air to underwater superoleophobicity (or underoil superhydrophobicity) by prewetted with water (or oil) [30].

Cellulose aerogels modified by organic materials (such as polymers, biopolymers, small molecules) have emerged as a fascinating class of oil absorbers. Polyvinyl alcohol (PVA) is typically used as the reinforcing agent of cellulose-based aerogels for improving their mechanical properties via physical crosslinks. PVA featuring with excellent water solubility, biodegradability, and biocompatibility, and inexpensive, have been used to reinforce the mechanical properties of cellulose-based aerogels by physical crosslinks. By using cross-linked PVA to reinforce the lightweight CNFs, Chhajed et al. prepared a porous nanofibrillated cellulose/PVA aerogel with superior mechanical property, low density, and excellent recyclability [106]. The sugarcane fiber/PVA aerogel reported by Thai et al., exhibited high porosity, ultra-flexibility, ultra-low density, which attributed to the lightweight of cellulose and the superior mechanical property of PVA [73]. With the assistance of the PVA, Gong et al. constructed a hydrogen bond linked CNC network and obtained a compressible CNCs/PVA aerogel [87]. Chitosan (CS) is the second abundant biomass material, featuring with inexpensive, biodegradable, and eco-friendly. In order to retain the three-dimensional (3D) structure of cellulose nanofiber skeleton, Zhang et al. adopted CS to prevent the shrinkage and strength the structural stability for achieving a CNF/CS composite aerogel with mechanical stability [138]. Using the structural advantage of porous biochar microparticles, Chen et al. fabricated a porous biochar/poly(vinylidene fluoride)/SiO${}_{2}$ hybrid nanofiber aerogel with a superhydrophobic surface and hierarchical porous structure. The separation efficiency of the-synthesized aerogel for micro and nano-scale water/oil emulsions can up to 99.6% [139]. A cellulose/tannic acid/castor oil aerogel was prepared by co-precipitating tannic acid and castor oil-based siloxane on the surface of cellulose nanofiber, showing a high adsorption capacity for various organic solvents and oils (53.2–113.8 g g${}^{-1}$) [140].

**Figure 9:**
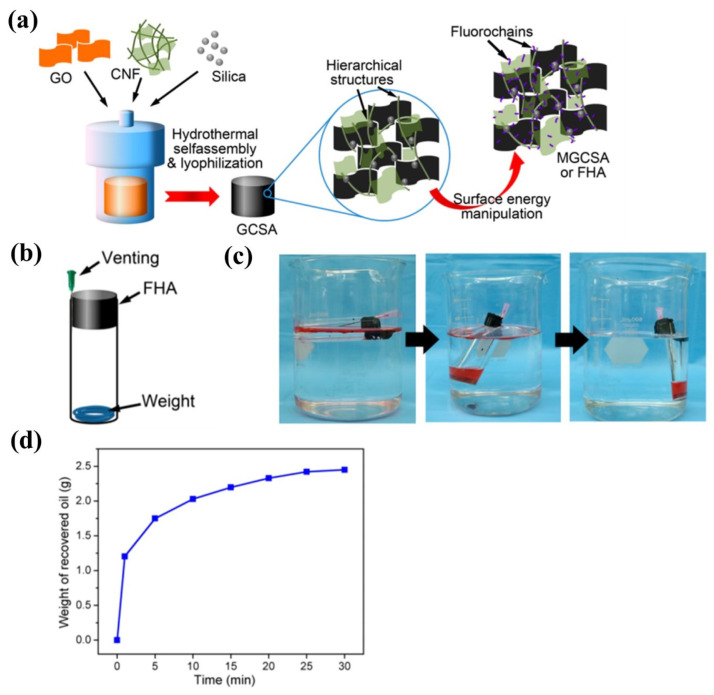
(**a**) Schematic illustration of the GO/CNF/Si aerogel (or FHA) fabrication process. (**b**) Illustration of the device developed for oil collection. (**c**) Photos demonstrating the oil collection process. (**d**) Weight of recovered oil over time with the developed device in (**b**). Reproduced with permission [91]. Copyright 2018, American Chemical Society.

Considering the unique nanostructure and ultra-small size of nanomaterials, researchers tried to incorporate various nanomaterials into cellulose-based aerogels for different purpose. He et al. utilized SiO${}_{2}$ fibers and polyacrylonitrile nanofibers to form hierarchical fibrous three network structure, resulting in the achievement of super-elastic aerogels. Due to its super-elasticability, the absorbed oil can be easily recovered by mechanically squeeze [141]. To manipulate the hierarchical structures and surface energy, Mi et al. combined CNFs with graphene oxide (GO) and silica nanoparticles for the fabrication of a superoleophilic hybrid aerogel (FHA) with extraordinary absorption capacity and efficiency (as shown in Figure 9) [91]. In Li’s work, the deposition of copper nanoparticles on the surface of cellulose fiber aerogel can directly achieve superhydrophobicity without further modification [116].

Nowadays, the strategy of the removal of oil by an external magnetic field has aroused increasing interest for researchers. Fe${}_{3}$O${}_{4}$ is the most used magnetic materials for designing oil sorbents with magnetic response, which has been successfully applied for the modification of cellulose sponges [79] and cellulose aerogels [108]. Xu et al. fabricated Fe${}_{3}$O${}_{4}$ NP/PVA/CNF hybrid aerogels, awarding the superior properties of high porosity (>98%), low density (13.84 kg m${}^{-3}$), high selectivity, and good magnetic response [37].

**Figure 10:**
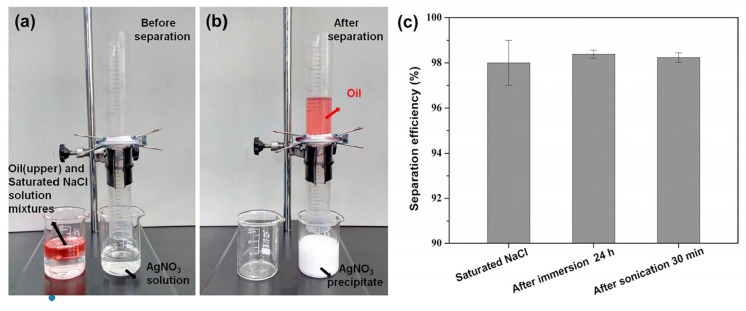
(**a**) The pre-wetted mesh was fixed between two plastic tubes. The mixture of oil (hexane) and saturated NaCl solution stayed layered before the separation. An aqueous AgNO${}_{3}$ solution was placed in the bottom beaker to detect Cl${}^{-}$ in the water phase. (**b**) Photograph showing the collected saturated NaCl solution and blocked oil(hexane) after the separation. The NaCl solution could react with AgNO${}_{3}$ solution to generate white AgCl precipitate immediately. No obvious, red-colored oil was observed in the AgCl precipitate. (**c**) Separation efficiency of the mesh for the separation of oil/water mixtures in highly salty environment, after immersion in saturated NaCl solution for 12 h, after sonication for 30 min, respectively. Reproduced with permission [142]. Copyright 2018, Elsevier Ltd.

Recently, several studies about cellulose-based hydrogels as coating materials have been reported. By coating cross-linked cellulose hydrogel on stainless mesh, it can successfully obtain superhydrophilic and underwater superoleophobic separating membranes. Ao et al. [142] and Xie et al. [31] respectively prepared cellulose-based hydrogel by adopting cellulose synthesized with different approach, which exhibited different compressive strength and separation performance. The cellulose hydrogel-coated mesh fabricated by Ao et al. through dip-coating and heating process (as shown in Figure 10) presented a separation efficiency of larger than 98.9% and a permeate flux of larger than 12,885 m${}^{-2}$ h${}^{-1}$ (solely driven by gravity).

Due to the inherent hydrophilicity of cellulose, further chemical modification is needed for most of reported hydrophobic cellulose-based materials, which may bring negatively influence on the biocompatibility of biomass-based sorbents. Despite it can prepare hydrophobic cellulose-based oil sorption materials via one-step direct approach, the cost during the whole process and the performance of materials are still need to consideration.

### 4.2. Chitosan

Chitosan as the second most abundant biopolysaccharide in nature, is usually collected from crab shells, lobsters, and so forth. CS is one type of non-toxic, biocompatible, biodegradable, and renewable material. Furthermore, it contains active amino and hydroxyl groups, which can easily hydrophobic modification for oil adsorption application (Table 5). Su et al. used sodium tripolphosphate/citric and octadecanethiol to modify chitosan sponge for achieving the superhydrophobicity [109].

It is desirable to combine CS with other materials for achieving special properties. Wang et al. utilized nanofibrillated cellulose to reinforce the chitosan matrix and fabricated bionanocomposite foams with excellent mechanical properties and thermal stability [143]. Magnetic nanoparticles have been introduced into the chitosan matrix for endowing magnetic response and then applying for the receival of oil via magnetic separation technology [144]. Quaternized chitosan decorated with Fe${}_{3}$O${}_{4}$ NPs was prepared by Zhang et al. which presented excellent separation efficiency of the oil from water at different pHs [84].

**Figure 11:**
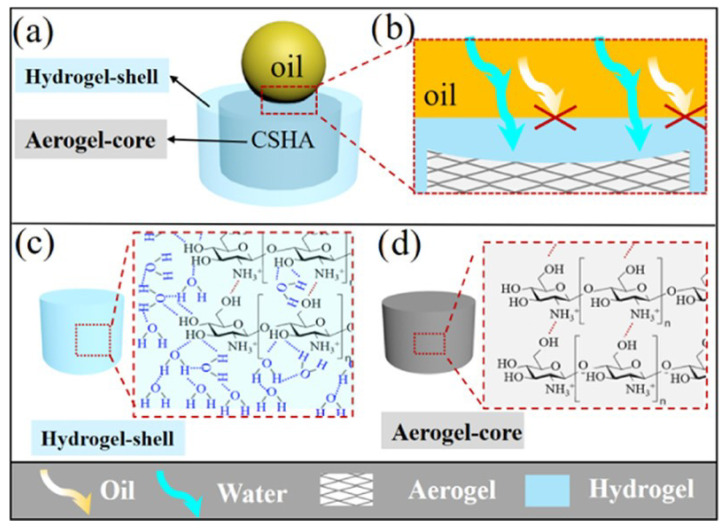
Mechanism of water selectively collected in CSHA: (**a**) The CSHA with the core/shell-like structure. (**b**) The hydrogel shell of the CSHA forbids further absorption of oil molecules, while water molecules are still able to penetrate the aerogel core until all pores are saturated. (**c**) Many water molecules absorbed on the surface of the CSHA and hydrogen bonds formed in and between molecules (red and blue dashed lines). At the same time, the CS aerogel is translated into the hydrogel. (**d**) Schematic of the aerogel core, that hydrogen bonds (red dashed lines) as the primary intermolecular interactions. Reproduced with permission [145]. Copyright 2020, American Chemical Society.

Chitosan-based aerogels: By various chemical modification, hydrophobic/oleophilic chitosan-based aerogels have been successfully fabricated and applied for spill oil cleanup. However, their oil absorption capacity, recycling ability, and oil-water selectivity highly depend on the fabricating techniques, hydrophobic modification agent, and micro-nano structure [38,76]. Zhang et al. prepared a porous core/shell structure aerogel with high porosity (>98.16%) and low density (10.19–36.05 mg cm ${}^{-3}$) by using CS aerogel as core and CS hydrogel as the surface coating (as shown in Figure 11) [145]. Cao et al. designed and constructed a chitosan aerogel with oriented wave-shaped layer microstructures, showing superelasticity and hydrophobicity [89].

**Table 4:** Cellulose based sorbents for spill oil cleanup.

Materials	Adsorption/Separation Performance	Recovery, Cycle	Refs.
Cellulose nanocrystals	35,000 ${\mathrm{Lm}}^{-2}$ $h^{-1}$ water flux >97% separation efficiency	No water flux declen after 12 >95% after 40	[28] [127]
Ethyl cellulose/carboxymethyl cellulose/${\mathrm{Fe}}_{3}O_{4}$	92.76% separation efficiency	>95% after 5	[128]
Cellulose pulp gelator	25–55 times of its own weight	-	[129]
Nanocellulose sponge	90–188 times of its own weight	Squeezing, 94% after 10	[36]
Cellulose acetate membrane	>99% separation efficiency	>98% after 10	[101]
Cellulose fibrous/expanded graphite foam	8–24 g $g^{-1}$	Squeezing, 60% after 15	[107]
Cellulose nanofibrils/natural rubber latex foam	13–42 g $g^{-1}$	Squeezing, 48–93.5% after 20	[29]
Cellulose microcrystalline/chitosan membrane	98.6% separation efficiency	-	[132]
Cellulose nanofiber sponge	>99% separation efficiency	-	[133]
Pinus elliotii based aerogel	13.73–19.55 g $g^{-1}$	-	[75]
Cellulose nanofiber	78.8–162.4 g $g^{-1}$	Squeezing, >99% after 20	[52]
Bamboo leaf based cellulose nanofiber	160–273 times of its own weight	-	[135]
Eichhornia crassipes/PVA aerogel	60.33–152.21 g $g^{-1}$	Squeezing, 66.33% after 16	[136]
Recycled waste cellulose nanofibrils aerogel	65–205 g $g^{-1}$	Squeezing, 80% after 30	[137]
Cotton fiber aerogel	19.8–41.5 times of its own weight	Vacuum filtration, >99% after 18	[81]
Lignocellulosic fiber	>99% separation efficiency	>99% separation efficiency after 50	[30]
Nanocellulose/${\mathrm{Fe}}_{3}O_{4}$ aerogel	33.24–68.06 g $g^{-1}$	-	[106]
Sugarcane bagasse/PVA aerogel	25 times of its own weight	-	[73]
Cellulose nanocrystals/PVA	21.2–32.7 times of its own weight	Squeezing, 88.5% after 10	[87]
CNF/tannic acid/castor oil aerogel	53.2–113.8 g $g^{-1}$	>55% after 10	[138]
Bacterial cellulose/${\mathrm{SiO}}_{2}$ aerogel	8–14 g $g^{-1}$	Squeezing, 88% after 1	[140]
Porous biochar/nanofibrous aerogel	118.5–120.3 g $g^{-1}$	Squeezing, 84% after 5	[139]
Cellulose nanofibris	38–68 g $g^{-1}$	Heating, 92-95% after 10	[91]
Cellulose/GO/silica NPs aerogel	67.8–164.5 g $g^{-1}$	Heating, >90% after 10	[116]
Ethyl cellulose/${\mathrm{SiO}}_{2}$ sponge	37.8 g $g^{-1}$	Heating, 87.6% after 50	[79]
CNF/PVA/${\mathrm{SiO}}_{2}$ aerogel	59–136 times of its own weight	-	[37]
Cellulose hydrogel/Wire mesh	98.9% separation efficiency	98.2% separation efficiency after 60	[142]
Cellulose hydrogel/Stainless mesh	>99% separation efficiency	98.9% separation efficiency after 10	[31]

Additionally, the preparation of composite aerogels based on chitosan and polymers (or other nanomaterials) have been widely investigated. By using fluorinated polydopamine to connect reduced graphene oxide and chitosan, Cao et al. fabricated a super-amphiphilicity in air and superoleophobicity underwater composite aerogel [110]. By adding nanofibrillated cellulose into chitosan matrix, a novel aerogel was developed with excellent mechanical stability, salt-tolerant, and superoleophobicity [32].

### 4.3. Bio-Carbon Based Materials

The distinctive properties of carbon-based materials, such as highly porose, larger specific surface area, low density, and excellent stability, makes it favorable for the sorption and separation of oil from water. Moreover, biomass materials containing abundant carbon elements can be carbon sources of carbon -based materials, endowing the environmentally friendly and economical. In recent years, biomass carbon materials with various structures have been extensively studied as sorbents for the spill oil treatment and recover (Table 6). That’s because biomass is rich in carbonaceous element and can easily carbonized at high temperature. Rao et al. selected the lignin as a carbon source to develop continuous 3D carbon foams due to the advantages of cheap, renewable, non-toxic and biodegradable [50].

**Table 5:** Chitosan based sorbents for spill oil cleanup.

Materials	Adsorption/Separation Performance	Recovery, Cycle	Refs.
Chitosan/sponge	23-60 g $g^{-1}$	Squeezing, 80% after 10	[109]
Nanofibrillated cellulose/chitosan foam	-	-	[143]
Chitosan/${\mathrm{Fe}}_{3}O_{4}$/${\mathrm{SiO}}_{2}$	98% separation efficiency	-	[84]
Chitosan/aerogel	34.1–54.2 g $g^{-1}$	-	[38]
Chitosan-acetic acid/aerogel	31–63 times of its own weight	Squeezing, >99% after 10	[76]
Chitosan-hydrogel/chitosan aerogel	99% separation efficiency	Extraction, >99% after 20	[145]
Unidirectional chitosan aerogel	53–117 times of its own weight	Squeezing, 94.17% after 50	[89]
Chitosan/r-GO/polydopamine	12–21 times of its own weight	Extraction, >90% after 11	[110]
Chitosan/nanofibrillated cellulose aerogel	>99% separation efficiency	Heating, 98% after 40	[32]

Carbon-based aerogel: Due to the efficient oil recovery and good reusability/recyclability of materials, a considerable amount of carbon aerogels derived from biomass materials have been investigated. In terms of raw materials, celluloses and their derives are easily carbonized directly and source abundant, which is the first choice for the fabrication of carbon-based aerogels. Yang et al. fabricated carbon aerogel through directly carbonization of typha orientalis, which demonstrated superior superhydrophobici/superoleophilic properties, compressibility (support pressure at 80%), high oil sorption capability (42–160 g g${}^{-1}$) and separation efficiency (99%) [39]. By using the same high-temperature carbonization method, Li et al. prepared poplars catkins microfibers-derived carbon-based aerogels with high absorbency (80–161 g g${}^{-1}$), high compressibility (80%), and ultralow density (4.3 mg cm${}^{-3}$). The tubular structure of poplars catkins microfibers contributes to the formation of a stable hollow 3D network carbon aerogel after carbonization and the achievement of excellent performance for oil sorption [146]. By make full use of the finger-like micro-structure and highly porosity of Liquidambar formosana, Feng et al. prepare porous carbon aerogel with good recyclability and high recovering efficiency for oils (99%) [147]. Liu et al. adopted sisal leaves with interconnected 3D and porous structure as the carbon source and prepared an elastic and ultralight carbon aerogel with superior absorption capacity, excellent oil absorption properties, and stability [148]. Combing the method of freeze-drying and pyrolysis, Yuan et al. prepared a novel carbon aerogel from bamboo pulp fibers. The as-synthesized aerogel displayed an ultralow density (5.65 mg cm${}^{-3}$), large specific surface area (379.39 m${}^{2}$ g${}^{-1}$), and extraordinary absorption capacity for various oils and organic solvents (50–150 g g${}^{-1}$) [149]. Zhang et al. treated plant leaves (premna microphylla leaves and sisal leaves) with NaClO${}_{2}$ and NaOH for the preparation of cellulose and then fabricated carbon aerogel via freeze drying/pyrolysis approach. The as-prepared aerogel presented 3D interconnected network structure with a specific surface area of 475.68 m${}^{2}$ g${}^{-1}$, and exhibited robust superhydrophobicity (water contact angle of 158°) for efficient absorption of oils from the water (77.7–147.3 g g${}^{-1}$ capacity for various oils) [150]. According to the above discussion, it can be concluded that the surface wettability and oil adsorption properties are different even though both are carbon aerogel synthesized from cellulose containing biomass materials. Additionally, the surface hydrophobicity, specific surface area, density, as well as oil absorption capacity strongly depends on carbon sources, the preparation method of celluloses and the micro-nano structure of final products.

Other cellulose containing materials (vegetables, crop wastes, fruit wastes, etc.) featuring with low cost and abundant sources, has also been applied for the preparation of carbon aerogels with high oil adsorption capacities, such as cabbage [151], corn bracts [152], banana peel [153], peanut shells [154], and so forth. In Zhu et al.’s work, pomelo peels containing rich celluloses and various functional groups were selected as the carbon source for fabricating interconnected 3D porous carbon aerogels with high specific surface area (466–759.7 m${}^{2}$ g${}^{-1}$) [19]. Taking advantage of the interconnected macropore network structure of durian shells, Wang et al. manufactured a carbon aerogel with good oil absorptive effect due to its excellent hydrophobicity and high specific surface area of 734.96 m${}^{2}$ g${}^{-1}$[155]. Cai et al. adopted cabbages as a natural nitrogen-doping agent and carbon source for the preparation of nitrogen-doped porous carbon aerogels. The obtained aerogel demonstrated 3D network and unique hierarchical structure, resulting in extraordinary performance in the application of retrieval of crude oils [151]. In order to conform hierarchical structure, Zhai et al. treated the starch by combing the template method and activation method and then obtained a ultrahigh specific surface area of 2367 m${}^{2}$ g${}^{-1}$ carbon aerogel [156]. By changing the original complex activation process to simple alkalization (KOH activation), Yue et al. prepared carbon aerogels based on rice straw with numerous micro-/meso- porose (porosity of 98.2%) and hierarchical structure (specific surface area of 1531 m${}^{2}$ g${}^{-1}$) contributing to the superior compressibility [65]. Differently, Cao et al. incorporated banana peel into waste papers matrix for the formation of porous and hierarchical structure under simple freezing process and the obtained carbon aerogel exhibited excellent compressibility and hydrophobicity/under-air superoleophilicity [153]. Besides, Dan et al. reported that the modification agent of NH${}_{4}$H${}_{2}$PO${}_{4}$ for the achievement of rough and hydrophobic properties on the Enteromorphoa-derived carbon aerogel surface [120]. In summary, the material properties of original carbon sources and the fabricating method, and the as-prepared aerogel micro-/nanostructure (such as hierarchical structure, porous, and rough surface, etc.) are both the designing and considering points of efficient bio-carbon aerogels.

**Figure 12:**
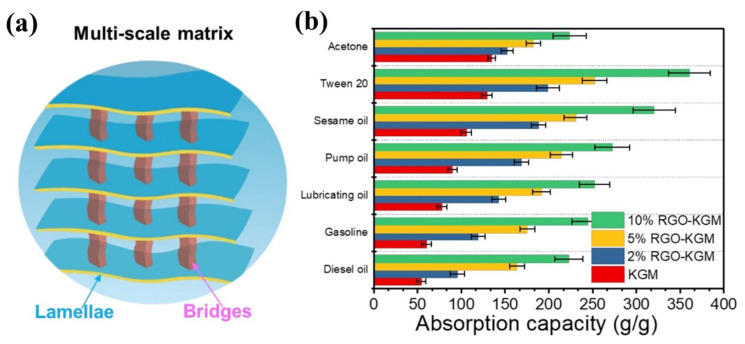
(**a**) The schematic of the as-prepared RGO-KGM carbon aerogel with mineral bridge structure. (**b**) Absorbency of the KGM and (2%, 5%, 10%) RGO-KGM carbon aerogels for various oils and organic liquids in the oil system. Reproduced with permission [159]. Copyright 2020, American Chemical Society.

Traditional carbon nanomaterials, such as graphene and its derives, carbon nanotubes, etc. have been widely used to combine with biomass materials for designing composite carbon-based aerogel due to their large specific surface area, as well as high mechanical and thermal stability. The strategy of using graphene oxide (GO) to improve the hydrophobicity and mechanical property of oil sorbents has been extensively utilized. As we all known, bio-carbon materials derived from different biomass materials own various micro-/nanostructure. Combining their own superior properties of bio-carbon materials (diverse micro-/nanostructures) and GO (great mechanical stability and hydrophobicity), researchers have done several interesting studies. For example, Meng et al. selected the high-active lignin as the carbon sources and GO as the mechanical enforcing material to construct carbon aerogels with extraordinary superhydrophobicity and mechanical property [117]. Luo et al. adopted 3D bacterial cellulose and 2D few-layer reduced graphene oxide (rGO) to fabricate robust carbon aerogel with excellent oil sorption performance [157]. Gu et al. selected high porose cotton fiber and rGO for the preparation of carbon aerogels with distinctive hydrophobicity, as well as mechanical stability [158]. Chen et al. utilized konjac glucomannan and rGO to fabricate carbon aerogel with high specific surface area, hydrophobicity, elasticity, and high oil sorption capacity thanks to the formation of unique stacked mineral bridge structure (as shown in Figure 12) [159]. Even though the introduction of GO into the bio-carbon materials can improve the mechanical property, the improved space is limited due to the disorder structure inside the carbon aerogel, which attributed to the strong Van der Waals force between GO nanosheets. Therefore, Xu et al. further improved the mechanical stability of cellulose-based carbon aerogel by adding PVA as the binder between cellulose nanofibers and GO, and the as-prepared carbon aerogel exhibited not only great oil-recovering and adsorption performance, but also excellent thermal and mechanical stability [160,161]. Except for graphene oxides, other materials with 3D network structure also were applied for the construction of high porose and high mechanical stable carbon aerogels. Take graphite nitride (g-C${}_{3}$N${}_{4}$) nanosheets (CNS) as one example, Ma et al. successfully synthesized a 3D superhydrophobicity carbon aerogel by selecting carbonized kapok fibers (CKF) as the 3D scaffold material and then dispersing porous CNS in the skeleton [162].

Inorganic nanoparticles with high surface area were utilized for decorating the carbon-based aerogel. By combining their own advantages of SiO${}_{2}$@MnO${}_{2}$ nanostructure and natural sisal cellulose, Yuan et al. obtained a bio-carbon aerogel composed of a hierarchical and interconnected network structure, displaying the superior properties of superhydrophobic, compressible, and high oil sorption performance [115]. Pryanka et al. synthesized starch derived zinc oxide carbon foam for the retrieval of crude oils [163].

**Table 6:** Bio-carbon based sorbents for spill oil cleanup.

Raw Materials	Aadsorption/Separation Performance	Recovery, Cycle	Refs.
Lignin	24 times of its own weight	burning, 95% after 7	[50]
Typa orientalis fibers	42–160 g $g^{-1}$	-	[39]
Poplars catkin microfibers	81-161 g $g^{-1}$	Heating, >99% after 10	[146]
Liquidambar formosana	2–2.90 g $g^{-1}$	Extraction, >96%after 10	[147]
Sisal leaves	90-188 times of its own weight	Squeezing, >86% after 10	[148]
Bamboo pulp fibers	510–150 times of its own weight	Extraction, >89% after 5	[149]
Sisal and premna microphylla	77.7–147.3 g $g^{-1}$	Extraction, >99% after 10	[150]
Corn bracts	77.67–143.63 times of its own weight	Squeezing, >90% after 10	[152]
Banana peel/wastepaper	35-115 times of its own weight	-	[153]
Peanut shells	27–50 g $g^{-1}$	Extraction, 94% after 9	[154]
Pomelo peels	5–36 g $g^{-1}$	Extraction, 72.15–98% after 5	[19]
Waste durian shell	3-19 g $g^{-1}$	Extraction, 75.31–95% after 5	[155]
Starch	36–45 g $g^{-1}$	-	[156]
Rice straw	29–33 times of its own weight	Heating, >99% after 5	[65]
Enteromorpha	62–140 g $g^{-1}$	Heating and extraction, >99% after 10	[120]
Lignin/GO	32.5–34 g $g^{-1}$	-	[117]
Bacterial cellulose/reduced GO	245–598 times of its own weight	Burning, 94.6% after 10	[157]
Cotton/reduced GO	16–27 times of its own weight	Burning, 98% after 10	[158]
Konjac glucomannan/reduced GO	54-360 times of its own weight	Burning, >99% after 10	[159]
CNF/PVA/GO	57–97 g $g^{-1}$	Burning, 51% after 10	[160]
Anisotropic GO/PVA/CNF	155–287 g $g^{-1}$	Burning, 90% after 10	[161]
Cellulose/${\mathrm{SiO}}_{2}$/${\mathrm{MnO}}_{2}$	60–120 g $g^{-1}$	Squeezing, 99% after 10	[115]
Kapok fibers/$g-C_{3}N_{4}$	28.3-58.1 times of its own weight	Heating, 85.3–90.1% after 5	[162]
Starch/ZnO	23–30 times of its own weight	Burning, 98.9% after 20	[163]
Popcorn	10–10.83 g $g^{-1}$	Heating, 65.7–93.6% after 5	[164]
Fe/egg yolk	28–78 times of its own weight	Heating, >99% after 6	[45]
Cotton balls	61–113 times of its own weight	Heating, >99% after 5	[93]
Bacterial cellulose	37–87 g $g^{-1}$	Heating, 71% after 5	[165]

**Figure 13:**
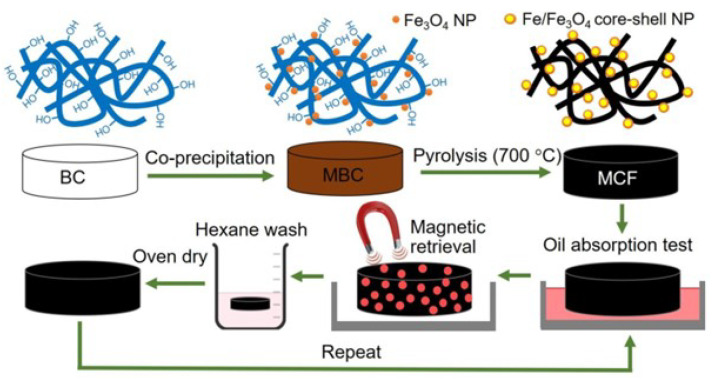
A diagram for the synthesis of magnetic carbon fiber (MCF) aerogels and the oil absorption measurement. Reproduced with permission [165]. Copyright 2020, American Chemical Society.

By using magnetic nanomaterials to fabricate nanocomposite biocarbon-based aerogels has drawn increasing interesting because it is easily and available to separate oil from the water via magnetic techniques. The magnetic nanomaterial, Fe, have been adopted in biomass-based carbon aerogel for simultaneously achieving the magnetic property and porose structure, such as popcorn [164], egg yolk [45], etc. Notably, the Fe/egg yok derived carbon aerogel made by Hossein et al. can be deposited on the sponge via simple dip-coating method for the application of oil-water separation [45]. By simply decorated with Fe${}_{3}$O${}_{4}$ nanoparticles on the surface of cotton-derived carbon aerogel, Lu et al. prepared superhydrophobic carbon aerogel with magnetism and rough surface contributing to the achievement of high sorption capacity for various organic solvents and oils (61–113 g g${}^{-1}$) [93]. As showing in Figure 13, Pimchanok et al. developed magnetic carbon aerogels by using bacterial cellulose as the carbon sources and decorating with Fe/Fe${}_{3}$O${}_{4}$ core-shell nanostructure, which exhibited good oil sorption property and high compressibility [165].

### 4.4. Natural Products

Biomass-based sorbents directly obtained from natural sources, such as plant fiber, fruits peels, etc., have demonstrated superior capabilities for the application of spill oil treatment because of their abundant, biodegradable, non-toxic, low cost, variety, and high content of active functional groups [36].

Raw materials: Cheaper, abundant, and biodegradable plant raw materials, such as cotton, kapok fiber, wood fiber, corn straw, rice husk, Luffa, and so on, have a loose fiber structure and numerous ample voids inside, which have been widely used as oil sorbents.

Kapok fibers features with large lumen and waxes covered fiber surface, resulting in their excellent buoyancy, good oleophilic and hydrophobic properties. Cao et al. compared the performance of oil wetting and oil sorption for three natural fibers (kapok, cattail, and cotton). They found that these three fibers showed quick oil uptake when applied as the oil sorbents [166]. Dong et al. utilized Kapok fiber to construct a depth filtering system, which can highly remove and recover oil from wastewater [167]. Zhang et al. prepared a superhydrophobic Kapok fiber with the absorption capacity range from 27.55 to 68.26 g g${}^{-1}$ [95].

Populous composed of lignocellulose was also applied for oil spill cleanup. In Yang et al.’s study, the acetylated lignin was used to modify Populus, which can significantly improve the maximum oil sorption capacity of this sorbent. (18.35 g g${}^{-1}$ after treated with 2% lignin concentration vs. 3.88 g g${}^{-1}$ without treatment) [121].

After studied the sorption performance of palm fibers for different oils, Ola et al. found that the oil adsorption capacity of palm fibers for different kinds of oil is both high [168]. Viju et al. pointed out to use nettle fibers as oil sorbents and its oil sorption capacity can be enhanced by grafting of butyl acrylate [10]. Mahmoud et al. investigated and compared the effect the effect on the oil adsorption performance for flax fibers based on two modification methods, acetylation process and microwave radiation. Their results indicated that acetylated flax fibers have higher oil sorption capacity (24.54 g g${}^{-1}$) than both raw (13.75 g g${}^{-1}$) and microwave treated fiber (17.42 g g${}^{-1}$) [68]. The milkweed fibers are hollow and their wall is very thin, allowing the sorbents based on these fibers possess good buoyancy properties. Panahi et al. prepared a superhydrophobic oil spill cleanup material by using milkweed floss fibers [169]. The obtained sorbents can absorb the crude oil of 100 g g${}^{-1}$ due to the superior capillary characteristics and hydrophobicity.

Some wasters, such as agricultural wasters (rice husk, corn straw, etc.), various fruit peels (such as banana, orange, cocoa, etc.) are rich in cellulosic content, and they are abundant as well as inexpensive, which is favorable to produce natural oil sorbents. Denirel et al. developed low-cost, environmentally friendly and efficient sorbent from four paper production industry wastes (bark, sawdust, belt filter waste, and rejects from the old paper mills) [170]. Akpomie et al. studied the effect on oil sorption performance of rice husk montmorillonite combo with different thermal treatment, and found that the sorption capacity increased from 5.8 (untreated) to 9.7 (treated) g g${}^{-1}$ [171]. Kamarudin et al. prepared green ceramic hollow fiber membrane by using corn cob ash waste and metakaolin for oil-water separation [22].

Alaa et al. investigated and found the different oil sorption capacity of banana peel-based sorbents under different conditions (oil types, sorption time, temperature, salinity, as well as materials surface properties) [172]. Gheriany et al. evaluated the oil sorption capacity of dried raw orange peel waste (OP) and thermally modified orange peel waste (TMOP) [57]. Compared to OP, the oil sorption capacity increased by 18–40% and the water uptake was significantly improved. Other wasters, like oil palm empty fruit bunch and cocoa pod [66], sugarcane bagasse [122], and Luffa [103], were also used as oil sorbents after chemical treatment and modification.

**Figure 14:**
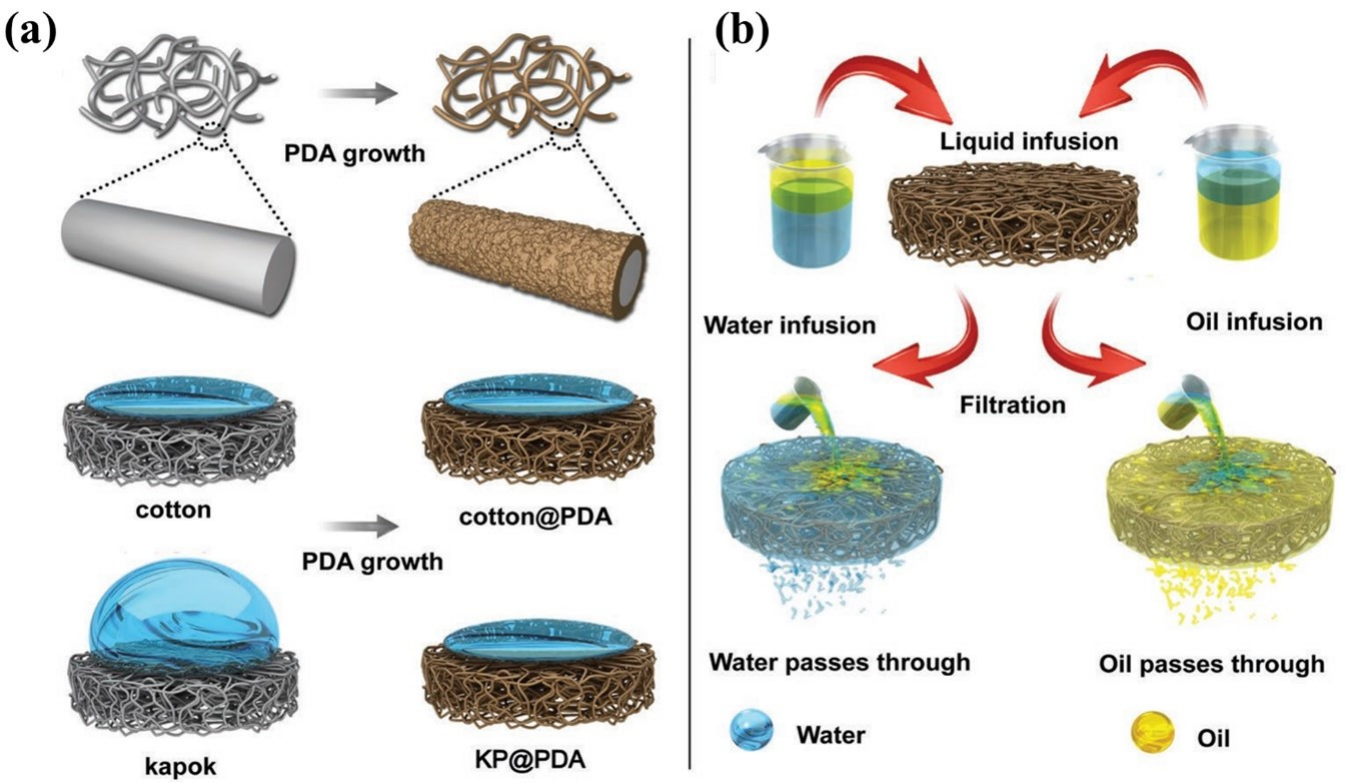
Schemes illustrate (**a**) the fabrication of PDA modified cotton fabric and kapok fabric and (**b**) the application of modified fabrics to selectively separate oil and water. Reproduced with permission [173]. Copyright 2020, Wiley.

Furthermore, various modifications to the natural materials have been investigated for the improvement of oil sorption performance and hydrophobicity. To incorporate nanoparticles, polymer, or their blends on the surface of natural materials before the addition of hydrophobic moieties is one strategy for hydrophobic modification of natural materials. Wang et al. constructed zinc oxide (ZnO) nanoneedles on kapok fiber surface to prepared a superhydrophobic fiber, which showing the sorption capacities of 40.8 to 70 g g${}^{-1}$ for various organic solvents and oils [59]. Later, this group transferred this ZnO nanomaterials to cotton fiber for the fabrication of durably superhydrophobic and superoleophiilc fiber, which exhibited a sorption capacity for various oils ranging from 29.5 to 52.8 g g${}^{-1}$[58]. Xu et al. attached (Heptadecafluoro-1,1,2,2-tetradecyl) trimethoxysilanepda-modified SiO${}_{2}$ nanoparticles onto the corn straw fiber surface and successfully achieved the superhydrophobicity/superoleophilicity [94]. By adjusting the growth behavior of polydopamine nanoparticle on cotton and kapok fibers surface, Mai et al. successfully obtained a dual-superlyophobic membranes with high separation efficiency and high flux (as shown in Figure 14) [173]. Zhang et al. prepared cotton-based porous materials with very high oil up-taking capacity for different kinds of oils thanks to the Synetic effect of beeswax and lignin on the cotton surface modification [123]. Zhang et al. combined mechanical stable polyurethane with 3D network structured Juncus effuses to the preparation of composite film with excellent oil absorption performance [174].

**Figure 15:**
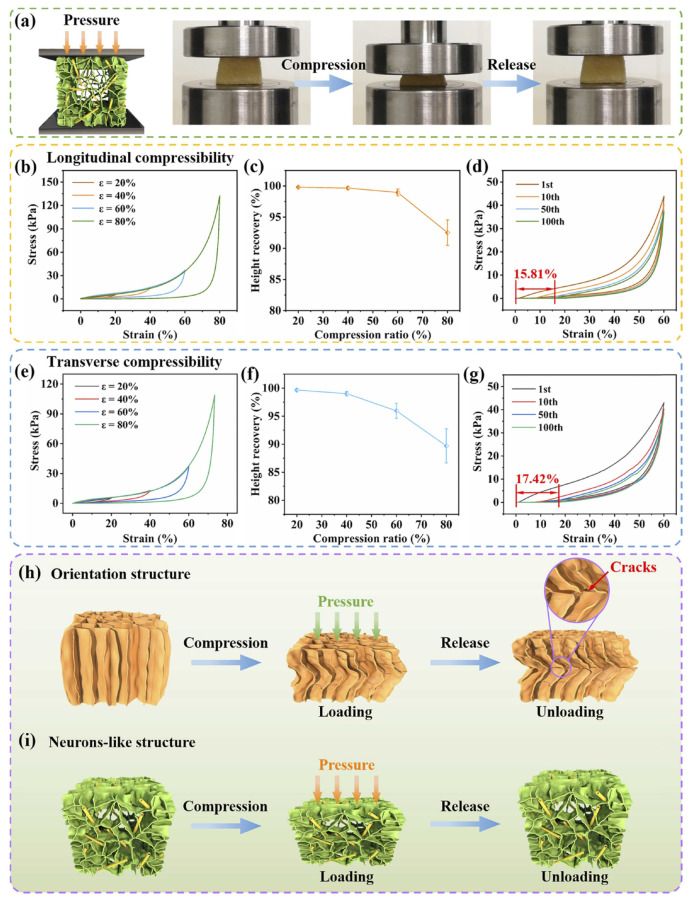
(**a**) The compression process of M-PCF/CS. Longitudinal compressibility: (**b**) Compressive stress versus strain curves for the M-PCF/CS with various maximum strains. (**c**) Height recovery ratio versus compression strain. (**d**) 100 cyclic compressions at a constant strain of 60%. Transverse compressibility: (**e**) Compressive stress versus strain curves for the M-PCF/CS with various maximum strains. (**f**) Height recovery ratio versus compression strain. (**g**) 100 cyclic compressions at a constant strain of 60%. Comparison of compression recovery performance between (**h**) materials with orientation structure and (**i**) M-PCF/CS with neurons structure. Reproduced with permission [177]. Copyright 2022, Elsevier Ltd.

Aerogels: Aerogels directly prepared from natural products were also applied for ideal oil sorption materials. By simple pretreatment of natural products which contains numerous celluloses, for example, corn stover [175], sugarcane bagasse [73], pomelo peel [74], and so forth, researchers fabricated various porous aerogels and composite aerogels with 3D porous structure. Cheng et al. prepared cotton aerogels and cotton/cellulose composite aerogels and found that the composite aerogels exhibited better oil sorption performance and mechanical stability than that of pure cotton aerogels thanks to their own distinctive characteristics of cotton and cellulose [72]. Bamboo fungus with hierarchical structures was adopted by Long et al. for the construction of tubelike and macropores structure aerogels, and the as-synthesized aerogel showed high sorption capacity and high selectivity [176]. To construct hierarchical structure, Deuber et al. utilized ultralight pullulan/PVA nanofibers to fabricate 3D aerogel and sponges via the process of electrospinning, freeze-drying, and in-situ thermal crosslinking [90]. Dong et al. achieved a robust superhydorphobic aerogel with tubular-lamellar interweaved structure by assembling chitosan on catkin fibers [177]. As shown in Figure 15, the resultant aerogels presented high porosity (96.12%), together with large longitudinal and transverse compressibility, as well as great oil sorption efficiency and capacity (28.8–78.1 g g${}^{-1}$). Notably, the compression behavior relates to the mechanical properties of sorbent materials, which is important for the oil adsorption from a practical application viewpoint.

Thanks to the advantages of abundant, biodegradable, and low cost for most natural products, sorbents directly derived from natural products are generally biodegradable, eco-friendly, and inexpensive. However, the downsides of these materials are poor buoyancy and low oil sorption capacity, that makes them typically needs additional modification.

### 4.5. Others

Apart from the above discussed biomass-based materials, other biomaterials and their derivatives were also reported for oil spill cleanup, such as lignin, alginate, protein, etc., (Table 7).

Lignin: Lignin has a complex three-dimensional network structure, which is the desirable structure for designing efficient sorbents. Yue et al. obtained a superhydrophobic and robust aerogel by taking full use of the merits of lignin and graphene. This ultralight and high porous aerogel exhibited extraordinary mechanical stability, sorption performance (adsorption capacity of chloroform is 102 g g${}^{-1}$) [40]. Yi et al. prepared a low-cost, ultralight, and high absorbent lignin/PVA aerogel [77]. The prepared aerogel showed high mechanical properties and relatively high absorption capacity. Zhang et al. prepared a porous super-hydrophobic sponge (water contact angle of 160°) by synergy of polydimethylsiloxane and lignin [98]. Cao et al. constructed a lignin-based multi-scale nanofibrous aerogel for the separation and recovering of oil from the water [178].

Alginate: Alginate is one kind of polysaccharide with linear chains and numbers of functional groups, which can easily form 3D network gel by interacting with metal ions. According to the study conducted by Wang et al., the hydrophobicity of sodium alginate (SA) foam can further be improved by crosslinked by Zr ions, which is attributed to the construction of microstructure on the material surface and the decreased surface energy after the modification of Zr ions [179]. Zhang’s group dispersed TiO${}_{2}$ NPs into the alginate matrix to improve the stability of SA-based aerogel under ultraviolet (UV) radiation [180]. Later, they further improved the mechanical stability by incorporating rGO into the as-prepared TiO${}_{2}$/alginate composite aerogel [33]. Finally, the separation efficiency, photocatalytic performance, and mechanical stability of this composite aerogel were both achieved the requirement for the practical application. Liu et al. prepared a novel porous biomass-based sponges by using zein and SA natural polymer materials and surface hydrophobic modifying with SiO${}_{2}$ nanoparticles and octadecanethiol [41]. Taking advantage of the hydrophobic property of SiO${}_{2}$ NPs and the superior mechanical property of GO, Yang et al. fabricated a superhydrophobic SA/GO/SiO${}_{2}$ aerogel with excellent selectivity, separation efficiency and oil sorption performance [51]. Another Yang’s group obtained a superelastic sponges with sandwich-like microstructure by growth SA flakes and GO nanosheets on the surface of Typha orientalis fibers, resulting in extraordinary mechanical compressibility and recoverability [181]. Tian et al. constructed an aerogel with oriented neurovascular network-like structure by utilizing tubular kapok fibers and SA, which exhibited durable superhydrophobicity, and excellent oil sorption and separation performance [182]. Besides, a new kind of peptide-polysaccharide aerogel by co-assembling of a simple dipeptide and the polysaccharide Konjac glucomannan [183].

**Table 7:** Other biomass based sorbents for spill oil cleanup.

Materials	Adsorption/Separation Performance	Recovery, Cycle	Refs.
Lignin/GO	42–102 g $g^{-1}$	Combustion, 90% after 20	[40]
Lignin/melamine/PVA	2–11 times of its own weight, >94% separtion	Extraction and distillation, 90% after 10	[77]
Lignin/cotton	36–46 times of its own weight	Extraction, >90.92%	[98]
Lignin/GO sheets/nanofibers	43–103 g $g^{-1}$	Squeezing, around 50% after 1	[178]
Alginate-Ca/Zr	11.2–25.9 g $g^{-1}$	Extraction, 50% after 6	[179]
Alginate/TiO_2_ NPs	98.7–99.7% separation efficiency	Reusing, 99.5% after 60	[180]
Alginate/TiO_2_/RGO	99.96% separation efficiency	69.5% after 60 and 30 min irradiation to recover	[33]
Alginate/nano-${\mathrm{SiO}}_{2}$	75.5–115.7% weight gain	53.7% after 1	[51]
Alginate/GO/${\mathrm{SiO}}_{2}$	17.92–43.92 g $g^{-1}$	Squeezing, 89.8% after 10	[41]
Alginate/Typha orientalis fibers/GO	98.2% separation efficiency	Squeezing, 90% after 20	[181]
Alginate/tubular Kapok fibers	29.6–62.8 g $g^{-1}$	Squeezing, 81-89.8% after 10	[182]
Peptide/polysaccharide Konjac glucomannan	30–180% weight gain	-	[183]
Gelatin/silica	2–27 times of its own weight	Heating, >99% after 10	[83]
Gelatin/${\mathrm{TiO}}_{2}$/PEI	99.72% oil rejection coefficient	-	[34]
Seeweed polysaccharide agar/genipin	>97% water rejection	-	[88]
Silk fibroin/polymethylsilsesquioxane	>2500 g $g^{-1}$	Squeezing, 99% after 6	[97]
Silk fibroin/sodium dodecyl sulfate	81.2–130 g $g^{-1}$	Squeezing, 89% after 10	[42]
Collagen/cellulose	20–60 times of its own weight	Burning, 95% after 7	[124]
Collagen	99.95% separation efficiency	99.9% separtion efficiency after 10	[69]
Collagen/ZIF-8	99.99% separation efficiency	Extraction, 78.63% after 6	[35]

**Figure 16:**
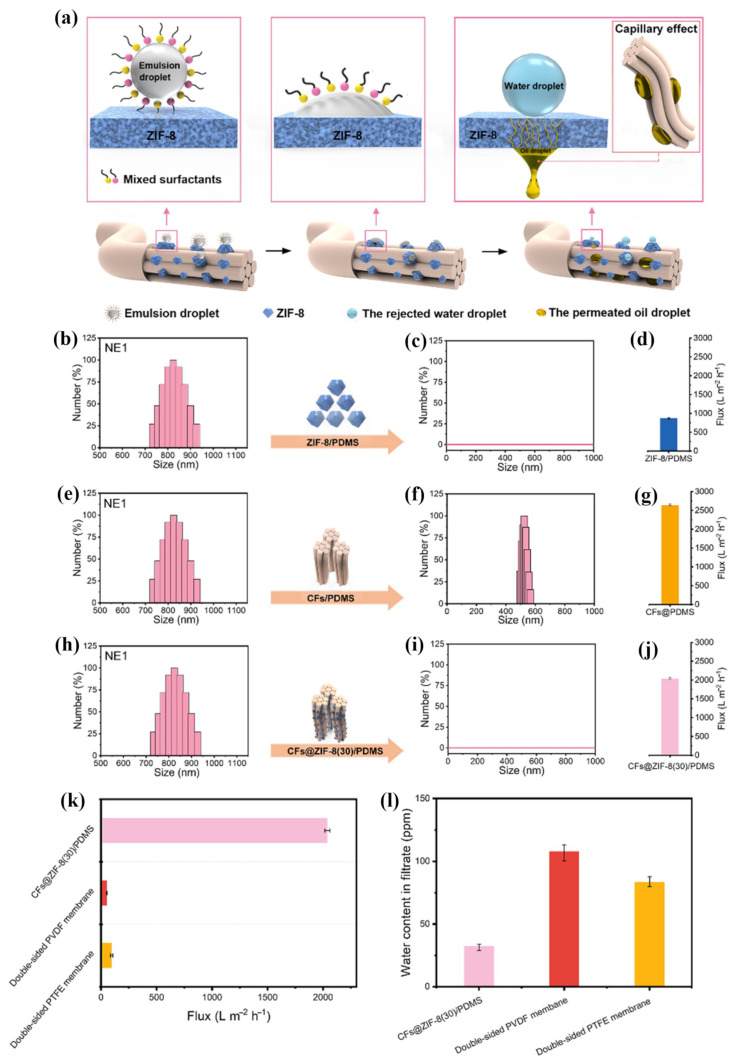
(**a**) Schematic illustration showing the proposed separation mechanism, DLS curves of NE1 (**b**,**e**,**h**) before and after the separation by (**c**) ZIF-8/PDMS, (**f**) CFs/PDMS and (**i**) CFs@ZIF-8(30)/PDMS, the corresponding fluxes of NE1 separated by (**d**) the ZIF-8/PDMS, (**g**) CFs/PDMS and (**j**) CFs@ZIF-8(30)/PDMS, (**k**) fluxes and (**l**) the corresponding water content in the filtrates of NE1 collected from the column packed with the CFs@ZIF-8(30)/PDMS, double-sided PVDF membrane, and double-sided PTFE membrane. Reproduced with permission [35]. Copyright 2020, American Chemical Society.

Protein: Protein and its derivatives is another important component to produce biomass-based oil sorbents. Yun et al. used gelatin with abundant reaction sites to reinforce the silica-based aerogel for the preparation of a silica-gelatin composite aerogel [83]. Jiang et al. fabricated a gelatin/TiO${}_{2}$/polyethyleneimine hybrid aerogels with hierarchical porous structure, superamphiphilic surface, and excellent oil/water separation properties [34]. Wang et al. prepared a porous lightweight bio-aerogels by introducing extensive microbubbles during gelation of gelatin [88]. Silk fibroin (SF) is a highly abundant fibrous protein-based polymer derived from the Bombyx mori silkworm cocoom. Maleki et al. proposed a new method to produce SF-based hybrid aerogel, featuring with compressible, lightweight, and hierarchically organized meso-/macroporous structure [97]. Vadodariya et al. prepared the protein functionalized aerogel membranes by using bovine serum albumin and naturally occurring genipin [42]. Zhang et al. developed an all-biomass hydrophobic aerogel by using Type I collagen as the matrix, dialdehyde carboxymethyl cellulose as the crosslinker and the mixed waxes as hydrophobic modifier [124]. Li’s group prepared amino-functionalized biomass-derived collagen fiber matrix with dodecyl hydrocarbon chains, featuring with favorable porosity, excellent hydrophobicity, high separation efficiency, and outstanding recycling performance [69]. Xiao et al. fabricated a composite fiber based on collagen fibers (CFs) and metal-organic frameworks (MOFs) with high separation response and efficiency for micro- and nano-emulsions (as shown in Figure 16). That’s because that the hierarchical structure of CFs can provide transport channels for oils and the microporous structure of MOFs can control the size of passing liquids [35].

## 5. Discussion, Future Perspectives, and Conclusions

### 5.1. Discussion

For the achievement of efficient oil absorption, biomass-based materials should have certain characteristics, such as good hydrophobicity and oleophilicity, high sorption capacity, buoyancy, recyclability, stability, and so forth. For those biomass materials for oil/water separation, the excellent selectivity and separation efficiency is also required. To improve the hydrophobicity of biomass-based materials, different surface modifications have been studied and displayed varying effectiveness and durability. However, environmentally friendly hydrophobic modification approaches for biomass-based materials are still expected and needed. Typically, the oil sorption capacity and oil/water separation efficiency are two important parameters considered for the design of biomass-based sorbents. While, except for sorption capacity (or separation efficiency), other factors, such as the sorption of high viscosity oil, superior mechanical properties, and cost (from materials to fabrication process) are also concerned for researchers.

### 5.2. Future Perspectives

For the further development of highly efficient biomass-based oil sorbents, several points still need to be concerned:

- Deeply understanding the sorption mechanisms. For materials with different surface chemistry and nanostructure, the oil uptake kinetics and mechanisms of sorption process may different, and it is also different when they under different working environmental conditions. Therefore, to efficient control and monitor the whole sorption process and achieve the durable application of sorbents, the kinetic and mechanisms of the oil sorption process under special environmental conditions, such as low temperature, strong acid, strong base, or high salinity, and so forth, still need to deeply investigate and understand.
- Developing environmental-friendly and cost-effectiveness modification technologies. It’s inevitable to do modification for the designed biomass-based materials so that to obtain superior wettability even though many researchers have done numerous efforts to prepare biomass-based sorbents via a straightforward and feasible approach. It is better to produce effective spill oil sorption materials with environmental-friendly, low-cost, and feasible modification methods without scarifies the superior properties of biomass materials.
- Designing high-performance and intelligent biomass composites. Typically, biomass-based materials are carbon-based small molecular or polymer, it is desirable to take the best advantage of different materials in various field for achieving highly efficient and intelligent oil sorbents.
- Promoting the mass and industrial production techniques. Currently, most of biomass-based sorbents have been fabricated in the laboratory and cannot be produced in large scale. The information and research relating to the mass production techniques for the industry is limited.

### 5.3. Conclusions

In summary, the recent progress of some biomass-based materials (cellulose, chitosan, bio-carbon, and natural products etc.) and their modification derivatives as sorbents for spill oil cleanup during the recent five years have been discussed in this review. To further improve the hydrophobicity and oil adsorption capacity, various modification methods have been broadly exploited that also summarized in this paper. Considering the severe damage to the survival of biological ecosystems and diversity by the release of spill oil, environmental-friendly and ecologically sustainable cleanup methods are urgently needed. Adopting biomass-based sorbent materials to remove and recover spill oils have shown the light prospectivity due to their unique features of eco-friendly, low-cost, effective waste management, undeniably. While, the development toward efficient, economic, environmental-friendly, sustainable, intelligent novel biomass-based oil sorbents will be the main research trend in the future.

## Figures and Tables

**Table 1:** Advantages and disadvantages of three types of sorbent materials.

Classification	Examples	Advantages	Disadvantages
Inorganic	Zeolites, silica	Availability, chemical inertness, non-flammable	Low capacity, poor selectivity, difficult recovery
Synthetic polymer	Polypropylene, polyurethane	Hydrophobic, moderate capacity, good reusability	Non-biodegradability, expensive, complex synthesis
Natural organic	Straw, peel	Abundant, inexpensive, biodegradability, eco-friendly	Low capacity, poor selectivity, poor hydrophobicity

**Table 2:** Basic information of cellulose, chitosan, bio-carbon based materials and natural products.

Material	Definition	Characteristic	Typical Examples
Cellulose	Long chain linear polysaccharide made of glucose (β-1,4), which can be extracted from plants, algae, tunicates and bacterial, etc.	Insoluble in water and general organic solvents, rich in hydroxyl groups, poor absorption capacity, rigid for natural cellulose	Lignin fiber, microfibrillated cellulose, cellulose nanocrystals, cellulose nanofibrils
Chitosan	Natural polysaccharide containing N-acetyl-D-glucosamine residue in its C2 position, which can be extracted from crab shells, lobsters, etc.	Contain numerous hydroxyl and amino groups, charge biopolymer, biodegradability, biocompatibility	Chitosan-based aerogel
Bio-carbon based materials	Carbon materials prepared by the carbonization of raw biomass materials at high temperature	Unique and various microstructure, high porosity, low apparent density, large specific surface area, high material purity, good stability	Cellulose based bio-carbon, three-dimensional nanostructured bio-carbon
Natural products	Products that are acquired directly from the nature without extraction or treatment	Multicomponent, versatile properties	Cotton, flax, wood fiber, corn straw, rice husk, fruit peel, luffa

**Table 3:** Material characteristics and examples for oil/water separation and oil adsorption.

Application	Material Characterizes	Example	Refs.
Oil/water separation	High porosity, high surface area, reusability, antifouling, high selectivity, tolerability, mechanical capacity, environmental-friendly	Cellulose nanocrystals	[28]
		Cellulose microcrystalline	[29]
		Lignocellulosic fiber	[30]
		Cellulose hydrogel	[31]
		NCF/chitosan aerogel	[32]
		Alginate/${\mathrm{TiO}}_{2}$/rGO	[33]
		Gelatin/${\mathrm{TiO}}_{2}$/PEI	[34]
		Collagen/ZIF-8	[35]
Oil sorption	high porosity, high surface area, mechanical stability, chemical stability, recyclable, environmental-friendly, low density, good buoyancy	Nanocellulose sponge	[36]
		CNF/PVA/${\mathrm{Fe}}_{3}O_{4}$ aerogel	[37]
		Chitosan sponge	[38]
		Chitosan/rGO aerogel	[39]
		Biocarbon aerogel	[40]
		Lignin/GO	[41]
		Alginate/GO/${\mathrm{TiO}}_{2}$	[42]
		Silk fibroin	[43]

## Data Availability

No new data were created or analyzed in this study. Data sharing is not applicable to this article.
